# Implication of Lateral Genetic Transfer in the Emergence of *Aeromonas hydrophila* Isolates of Epidemic Outbreaks in Channel Catfish

**DOI:** 10.1371/journal.pone.0080943

**Published:** 2013-11-20

**Authors:** Mohammad J. Hossain, Geoffrey C. Waldbieser, Dawei Sun, Nancy K. Capps, William B. Hemstreet, Kristen Carlisle, Matt J. Griffin, Lester Khoo, Andrew E. Goodwin, Tad S. Sonstegard, Steven Schroeder, Karl Hayden, Joseph C. Newton, Jeffery S. Terhune, Mark R. Liles

**Affiliations:** 1 Department of Biological Sciences, Auburn University, Auburn, Alabama, United States of America; 2 USDA, Catfish Genetics Research Unit, Stoneville, Mississippi, United States of America; 3 Department of Fisheries & Allied Aquacultures, Auburn University, Auburn, Alabama, United States of America; 4 Alabama Fish Farming Center, Greensboro, Alabama, United States of America; 5 Mississippi State University, College Veterinary Medicine, Stoneville, Mississippi, United States of America; 6 Aquaculture & Fisheries Center, The University of Arkansas at Pine Bluff, Pine Bluff, Arkansas, United States of America; 7 Bovine Functional Genomics Laboratory, USDA, ARS, Beltsville, Maryland, United States of America; 8 Department of Pathobiology, Auburn University, Auburn, Alabama, United States of America; Institut National de la Recherche Agronomique, France

## Abstract

To investigate the molecular basis of the emergence of *Aeromonas hydrophila* responsible for an epidemic outbreak of motile aeromonad septicemia of catfish in the Southeastern United States, we sequenced 11 *A. hydrophila* isolates that includes five reference and six recent epidemic isolates. Comparative genomics revealed that recent epidemic *A. hydrophila* isolates are highly clonal, whereas reference isolates are greatly diverse. We identified 55 epidemic-associated genetic regions with 313 predicted genes that are present in epidemic isolates but absent from reference isolates and 35% of these regions are located within genomic islands, suggesting their acquisition through lateral gene transfer. The epidemic-associated regions encode predicted prophage elements, pathogenicity islands, metabolic islands, fitness islands and genes of unknown functions, and 34 of the genes encoded in these regions were predicted as virulence factors. We found two pilus biogenesis gene clusters encoded within predicted pathogenicity islands. A functional metabolic island that encodes a complete pathway for *myo*-inositol catabolism was evident by the ability of epidemic *A. hydrophila* isolates to use *myo*-inositol as a sole carbon source. Testing of *A. hydrophila* field isolates found a consistent correlation between *myo*-inositol utilization as a sole carbon source and the presence of an epidemic-specific genetic marker. All epidemic isolates and one reference isolate shared a novel O-antigen cluster. Altogether we identified four different O-antigen biosynthesis gene clusters within the 11 sequenced *A. hydrophila* genomes. Our study reveals new insights into the evolutionary changes that have resulted in the emergence of recent epidemic *A. hydrophila* strains.

## Introduction

*Aeromonas hydrophila* is the causative agent of motile Aeromonad septicemia (MAS) of catfish [1]. MAS was not a disease of major concern for the catfish industry in the Southeastern United States until 2009 [2], when several commercial catfish operations in western Alabama experienced outbreaks of MAS resulting in industry-wide losses of food-sized catfish totaling over 8 million pounds [3]. Since 2009 this MAS epidemic has spread within the Southeastern United States, and cases have now been identified in Mississippi and Arkansas [2]. Experimental disease challenges have demonstrated that epidemic virulent *A. hydrophila* (VAh) isolates obtained from recent epidemic outbreaks in catfish are highly virulent to channel catfish compared to reference isolates of *A. hydrophila* (RAh) obtained from diseased fish from previous non-epidemic outbreaks [4]. 

Virulence factors of *A. hydrophila* including pili [5], hemolysin [6], serine protease [6,7], metalloprotease [8], cytotoxic enterotoxin [9,10], S-layer [11,12], and the type III secretion system [8] have been shown to be important for fish disease. Virulence factors of *A. hydrophila* are multifactorial and the concerted action of several factors are required to cause disease in fish, at least for previously described isolates [8,10]. Though a large number of *A. hydrophila* virulence factors involved in different fish diseases have been reported, most of their mechanisms of pathogenesis have yet to be studied, and no studies have evaluated the specific virulence factors within VAh strains. A PCR-based subtractive genomic hybridization approach has been used to identify epidemic-associated genes within VAh isolates [13]. However, this study did not provide comprehensive information on the genomic regions and virulence factors associated with VAh strains.

The emergence of infectious agents is frequently driven by the plasticity of bacterial genomes due to the loss and acquisition of foreign genetic elements [14]. Lateral gene transfer (LGT) by means of prophages [15,16], integrating conjugative elements [17] and plasmids [18] play significant roles in bacterial virulence. Prophages are well known for their ability to induce lysogenic conversion by introducing virulence genes [16] and changing the genome architecture by introducing genetic elements that increase fitness [15]. The rapid onset of the recent epidemic in catfish is suggestive of an emerging strain of *A. hydrophila* that has acquired new genetic elements via LGT.

Until now, none of the *A. hydrophila* strains obtained from fish have been subjected to whole genome sequencing. The complete genome sequence of *A. hydrophila* ATCC 7966 (obtained from a milk sample) has been determined, yet the nature of the pathogenicity of this strain has not been studied in fish [19]. Phylogenetic analysis and virulence studies have demonstrated differences between the VAh and RAh strains, with the VAh strains being at least 200 times more virulent than a RAh isolate obtained from a diseased catfish in 1998 [4]. This study was initiated to compare the genomes of highly virulent VAh isolates with that of RAh isolates and identify epidemic-associated genetic elements to reveal mechanisms fostering the hyper-virulence of these VAh strains. The molecular characterization of epidemic strains will provide the framework for the development of vaccines, therapeutics, and rapid diagnostics to facilitate the control of this emerging catfish pathogen. 

In this study we have sequenced the genome of 11 *A. hydrophila* isolates including 6 epidemic and 5 reference isolates using next-generation sequencing technology. Comparative analysis of these *A. hydrophila* genomes demonstrated that recent epidemic isolates are clonal and carry a large number of epidemic-associated unique genetic regions missing in reference isolates. This study provides detailed insight into the molecular evolutionary changes that have occurred in *A. hydrophila* epidemic isolates and suggests that the acquisition of novel genetic elements via LGT may have played a role in the emergence of this pathogenic strain. 

## Materials and Methods

### Ethics statement

All experiments conducted with vertebrate animals (catfish) were approved by the Institutional Animal Care and Use Committee (IACUC) review board at Auburn University in accordance with the animal welfare guidelines specified in the United States.

### Bacterial isolates

Based on the biochemical and molecular phylogenetic data, a collection of 11 *A. hydrophila* isolates ((n=6 epidemic *A. hydrophila* (VAh); n=5 historical "reference" *A. hydrophila* isolates (RAh)) were selected for sequencing (Table 1). All of the *A. hydrophila* isolates were identified by standard biochemical tests [20,21] and confirmed by sequencing of the B-subunit of the DNA gyrase (*gyrB*) gene [22]. Biochemical tests included Gram stain, motility, cytochrome oxidase, glucose utilization, resistance to 0/129, sucrose utilization, esculin hydrolysis, Voges-Proskauer, ornithine decarboxylase, DL-lactate utilization and urocanic acid utilization.

**Table 1:** Summary of draft genome sequences from 11 different *A. hydrophila* isolates obtained from diseased fish.

Strains	Source of isolates	Year of isolation	Type**^*a*^**	Contigs >200bp	N50 contig size (bp)	%G+C	Total bp in assembly	GenBank Accession no.
AL97-91	Diseased Tilapia	1997	RAh	111	159,889	61.2	4826834	SRX157795
TN97-08	Diseased Blue Gill	1997	RAh	94	144,878	60.8	5197004	SRX157873
MN98-04	Diseased Tilapia	1998	RAh	98	140,863	61.1	4863171	SRX157796
AL06-01	Diseased catfish	2006	RAh	122	120,531	61.3	4750603	SRX157912
AL06-06	Diseased Goldfish	2006	RAh	133	104,809	61.4	4844135	SRX157794
AL09-79	Diseased Catfish	2009	VAh	91	111,260	60.9	4975016	SRX157791
ML09-119	Diseased Catfish	2009	VAh	100	167,870	60.9	5003533	SRX157759
ML09-121	Diseased Catfish	2009	VAh	93	182,452	60.9	4998164	SRX157784
ML09-122	Diseased Catfish	2009	VAh	156	89,294	60.9	4979378	SRX157790
AL10-121	Diseased Catfish	2010	VAh	98	167,914	60.9	5010737	SRX157792
PB10-118	Diseased catfish	2010	VAh	100	143,368	60.9	5060794	SRX157793

Note:***^a^*** VAh and RAh stand for virulent *Aeromonas hydrophila* and reference *Aeromonas hydrophila*, respectively.

### Koch’s Postulates

To determine the etiologic agent of the recent epidemic outbreak of MAS in channel catfish, *A. hydrophila* ML09-119 isolated from kidney tissue of a diseased channel catfish was used for experimental challenge of catfish fingerlings in aquaria. *A. hydrophila* AL06-06 is a RAh strain obtained from a diseased fish but without any association with an epidemic outbreak was also used for challenging channel catfish. Catfish fingerlings were acclimated for 15 days prior to challenge with *A. hydrophila* ML09-119, AL06-06 or sham treatment. For each treatment, a group of ten fish was placed in a 52-liter tank with de-chlorinated water and for each treatment three different tanks were randomly assigned. Each of the fish received more than 1.0×10^6^ CFU of *A. hydrophila*, since catfish intraperitoneally infected with a similar dose usually kill more than 95% of challenged fish [4]. Each of the sham-injected fish received 100 µl of tryptic soy broth (TSB). Dead fish from each treatment group were subjected to necropsy and bacteria were isolated from liver tissues by plating them onto a tryptic soy agar (TSA) plate. Bacterial pure cultures were subjected to biochemical tests and used for a subsequent challenge experiment as described previously. Following challenge experiments, dead fish were necropsied and bacterial strains were re-isolated to confirm their identity.

### Phylogenetic analysis

Evolutionary relationships of 107 *A. hydrophila gyrB* gene sequences were determined by the construction of an unrooted phylogenetic tree using MEGA5 [23]. The evolutionary history was inferred using the Maximum Parsimony method [24]. Results from a maximum parsimony analysis of all 107 strain *gyrB* sequences were used to remove clades that were more distantly related to the VAh strains, while retaining all RAh strains, and 37 strains were re-analyzed by maximum parsimony analysis. The percentage of replicate trees in which the associated taxa clustered together in the bootstrap test (1000 replicates) is shown next to the branches. All positions containing gaps and missing data were eliminated from the dataset (Complete Deletion option). There were a total of 929 positions in the final dataset. The MP tree was obtained using the Close-Neighbor-Interchange algorithm with search level 3 in which the initial trees were obtained with the random addition of sequences (10 replicates). The codon positions included were 1st+2nd+3rd+Noncoding. All positions containing gaps and missing data were eliminated from the dataset (Complete Deletion option). 

### Sequencing, assembly and annotation

Barcoded Illumina libraries were prepared from each strain using a Nextera DNA Sample Prep Kit (Epicentre, Madison, WI). Equal amounts of library products were pooled, and paired sequences were obtained from an Illumina GAIIx sequencer using 150 bp read lengths (Illumina, Inc., San Diego, CA). Sequences from each library were de-convoluted and assembled *de novo* using ABySS v1.2.6 [25] on the Amazon Elastic Compute Cloud. A minimum of 10 paired reads was required to join unitigs into contigs. Multiple assemblies were produced per isolate using varied kmer settings, and 200 bp or larger contigs from the most contiguous assembly was further analyzed. In addition to Illumina sequencing, the VAh type strain ML09-119 was subjected to 454 pyrosequencing. A bar-coded phage DNA sublibrary was prepared at the Lucigen Corporation (Middleton, WI) and sequencing was conducted at Engencore (Univ. of South Carolina). The methods for the determination of prophage sequences are described in the SI methods. The reads from Illumina and 454 were *de novo* assembled using CLCBio Genomics Workbench (version 4.9). Gene prediction and annotation were carried out using GeneMark [26] and the RAST annotation server [27], respectively. 

### Identification of unique regions within the genome of ML09-119

*In silico* genomes for each of the reference isolates including AL06-01, AL06-06, AL097-91, MN98-04 and TN97-08 were constructed by force-joining each of their respective contigs. Each of the genomes were greater than 4.8 Mbp which was presumed as a near complete genome since the size of the only sequenced *A. hydrophila* ATCC 7966 is about 4.7 Mbp [19]. The *in silico* genome of AL06-06 was used as a scaffold to assemble trimmed, paired-end sequence reads of epidemic isolate ML09-119 using CLC Bio Genomic Workbench (v 4.9). The parameters that were used for each reference mapping was as follows: mismatch cost =2, insertion cost =3, deletion cost =3, length fraction =0.5 and similarity =0.9. The un-mapped (paired) reads of ML09-119 sequences from this reference mapping were then reference mapped against the force-joined contigs of AL97-91, and this process was repeated with the RAh strains AL06-01, MN98-04, TN97-08, and ATCC 7966. The un-mapped ML09-119 sequence reads that did not match with any of the five RAh strains or ATCC 7966 strain were considered as ML09-119-associated sequences that were uniquely present in strain ML09-119. To identify the distribution of those un-mapped reads in the genome of ML09-119, we conducted reference mapping of the un-mapped sequence reads to the *de novo* assembled genome of ML09-119 which was about 5.0 Mbp. The regions of the ML09-119 genome that were aligned with ML09-119 un-mapped reads were considered as ML09-119-associated unique regions. Those ML09-119-specific sequences were extracted manually for further analysis. Since later analyses determined that all of the genomic regions that were ML09-119-specific were also present within each of the other VAh strains, these regions are subsequently referred to as VAh-associated genomic regions. Each of the VAh-associated genomic regions was compared to a set of contigs available in GenBank for ML09-119 (Accession no. NC_021290) [28].

### Defining the Pan and Core genome

Conserved gene families within the genome of *A. hydrophila* isolates were identified according to the methods described as [29] that used ‘50/50’ rule for defining conserved protein families [30]. According to this rule two sequences are considered as a member of the single family if alignment between two proteins is 50% in a single span and contained at least 50% identities. The conserved gene families in the collection of *A. hydrophila* genomes were found by using the BLASTp algorithm for all of the proteins of each proteome against all the proteins of the query proteome using the microbial pan-genomics tool [31] kindly provided by David W. Ussery, at The Technical University of Denmark, Lyngby, Denmark.

### BLAST Matrix

The BLAST matrix algorithm was used for the pairwise comparison of the proteomes of each of the 12 *A. hydrophila* isolates to another according to the methods described by Friis et al [29]. This algorithm determines the percent similarities between two isolates by measuring the ratio of the number of conserved gene families shared between isolates to the total number of gene families in the isolates. The distribution of the conserved gene families within the genome of 12 *A. hydrophila* isolates was presented in a triangle-shaped matrix. 

### Prediction of Genomic Islands

The epidemic *A. hydrophila* strain ML09-119 genome sequences were subjected to genomic island prediction using IslandViewer [32], a computational tool that integrates three different genomic island prediction methods including IslandPick, IslandPath-DIMOB, and SIGI-HMM [32]. The concatenated contigs of ML09-119 strain consisted of ~ 5.0 Mbp nucleotides that were converted to GenBank format using the Sequin program (version 11.9). The GenBank formatted sequence file was uploaded to the IslandViewer web based tools for scanning the ML09-119 genome for the presence of genomic Islands. IslandViewer used three different *Aeromonas* species such as *A. hydrophila* *strain* ATCC 7966*, A. salmonicida* and *A. caviae* for the comparison of query sequences of ML09-119 provided for GIs prediction. To identify the epidemic-associated unique GIs, the nucleotide sequences of all the GIs in the ML09-119 strain predicted with IslandViewer tools were forced joined and used as a reference sequence to conduct a reference mapping against trimmed pair-end reads of all five RAh strains. The concatenated GI sequences that did not map with the sequence reads of RAh strains were considered as VAh-associated unique GIs. 

### Electron Microscopy

Concentrated phage particles obtained from a mitomycin C-treated *A. hydrophila* ML09-119 culture were negatively stain with 2% phosphotungstic acid (pH 6.5) after placing on 300 mesh formvar- and carbon-coated copper grids (Electron Microscopy Services, PA) for 15 minutes. The grids were examined at different magnifications to determine the size and morphology of phages using a Zeiss EM10 Transmission Electron Microscope (Zeiss, Germany).

### 454 pyrosequencing of induced prophage genome

Phage genomic DNA was extracted from concentrated phage lysates obtained from mitomycin C-treated *A. hydrophila* ML09-119 strain as previously described [33]. A bar-coded phage DNA sublibrary was prepared at the Lucigen Corporation (Middleton, WI) compatible with 454 titanium chemistry. A 1/8 plate sequencing run of the 454 pyrosequencer was conducted at Engencore (Univ. of South Carolina) that yielded 25,873,898 bp from 96,898 reads (267 bp average length) from the phage DNA library.

### Prediction of virulence factors in the epidemic-associated unique genomic regions

Virulence factors were predicted within the unique VAh-associated genome sequences using the Virulence Factors Database (VFDB) [34] which contains 2,353 proteins from different pathogenic bacteria as of March 2012. All of the proteins from the VFDB were retrieved and a local database was created in the CLC Bio Genomic Workbench. Predicted proteins encoded by genes within the unique regions were subjected to BLASTp analysis against the virulence factors database using CLC Bio Genomics Workbench to identify the occurrence of virulence factors associated with epidemic strains. An E-value threshold of 10^-10^ was selected to exclude proteins of distant homologs.

### Screening of *A. hydrophila* strains for VAh-associated genes by PCR

A PCR assay was used to test for the presence of VAh-associated genes within the genome of *A. hydrophila* cultured isolates. A VAh-associated gene whose presence was confirmed on the complete genome sequence of all six VAh strains, and had no significant BLAST hit against the GenBank nr/nt database was used for PCR screening of *A. hydrophila* cultured isolates obtained from diseased catfish, pond sediments, and fish samples taken from a processing plant. A multiplex PCR was carried out using primer pairs specific to the VAh-specific region C13R2 [35] and the *gyrB* gene. The *gyrB*-specific primers were used in the multiplex PCR to provide an internal control. Amplicons present for both pairs of primers were considered as positive for VAh specific isolates. On the contrary, amplicons present for *gyrB*-specific primers but absent for C13R2-specific primers indicated that this was an RAh isolate. Genomic DNA was extracted from *A. hydrophila* isolates according to the methods described previously [36]. One hundred ng of genomic DNA per 25 µl PCR reaction was used as a template for PCR amplification of VAh-associated genes using the following thermal cycling parameters: 94°C for 2 min, then 35 cycles of 94°C for 30 sec, 50°C for 30 sec, 72°C for 1 min, and a final extension at 72°C for 5 min. Type strain *A. hydrophila* ATCC 7966 was used as a negative control whose genome sequence does not possess any epidemic-associated genes [19].

### Evaluating the growth response of *A. hydrophila* strains for using *myo*-inositol as a sole carbon source

An isolated colony from a pure culture of an *A. hydrophila* isolate was used to inoculate a 2 ml TSB culture and was grown at 28°C overnight with shaking at 200 rpm. The cell suspension was pelleted by centrifugation at 10,000 × *g* for 10 min and then the cells were washed twice with 1× PBS buffer and re-suspended in M9 minimal medium supplemented with 5.5 mM of *myo*-inositol (M9I) to an OD_600_ of 0.5. The cell suspension was then serially diluted 1:100 in M9I and 100 µl of the 1:100 diluted cell suspension was used to inoculate 1.9 ml of M9I. Bacterial cultures were then grown for up to 144 hours and the OD_600_ was recorded at 24 hour intervals to determine the ability of each strain to use *myo*-inositol as a sole carbon source. *A. hydrophila* isolates ML09-119 and AL06-06 were used as positive and negative control, respectively, for the myo-inositol utilization assay.

### Nucleotide Sequence Accession Numbers

The *gyrB* gene sequences were deposited within the GenBank nr/nt database under the accession numbers JX275833 to JX275847. Illumina sequence reads were submitted to the NCBI Sequence Reads Archive (SRA) under the accession numbers SRX157795, SRX157873, SRX157796, SRX157912, SRX157794, SRX157791, SRX157759, SRX157784, SRX157790, SRX157792, and SRX157793.

## Results

### *A. hydrophila* ML09-119 isolate is the etiologic agent for the epidemic outbreak of MAS in channel catfish

To determine whether *A. hydrophila* ML09-119 was highly virulent in channel catfish, catfish fingerlings were intraperitoneally inoculated with more than 5.0×10^6^ CFU/fish. Fish were also challenged with *A. hydrophila* AL06-06, a reference strain obtained from disease fish but not from an epidemic outbreak. *A. hydrophila* ML09-119 killed approximately 80% of the fish within 24 hours, whereas 20% of the fish were killed by *A. hydrophila* AL06-06 by one week post-inoculation, and this was a statistically significant difference in mortality (P < 0.05) (data not shown). All of the dead catfish demonstrated clinical signs of disease caused by *A. hydrophila* and groups of fish that were injected with a sham treatment did not have any evident disease (data not shown). Bacteria were re-isolated from the dead fish after necropsy and their identity was confirmed as *A. hydrophila* as ML09-119 and AL06-06, respectively. The re-isolated bacteria were used for infecting new populations of catfish fingerlings. We observed a similar mortality rate and clinical symptoms specific to MAS caused by *A. hydrophila* in dead fish similar to the first challenge experiment (data not shown). Biochemical tests confirmed the identity of the re-isolated bacteria as *A. hydrophila*. These results demonstrated that *A. hydrophila* ML09-119 isolate fulfills Koch’s Postulates and is highly virulent in channel catfish.

### Phylogenetic analysis of *A. hydrophila* isolates

**Figure 1:**
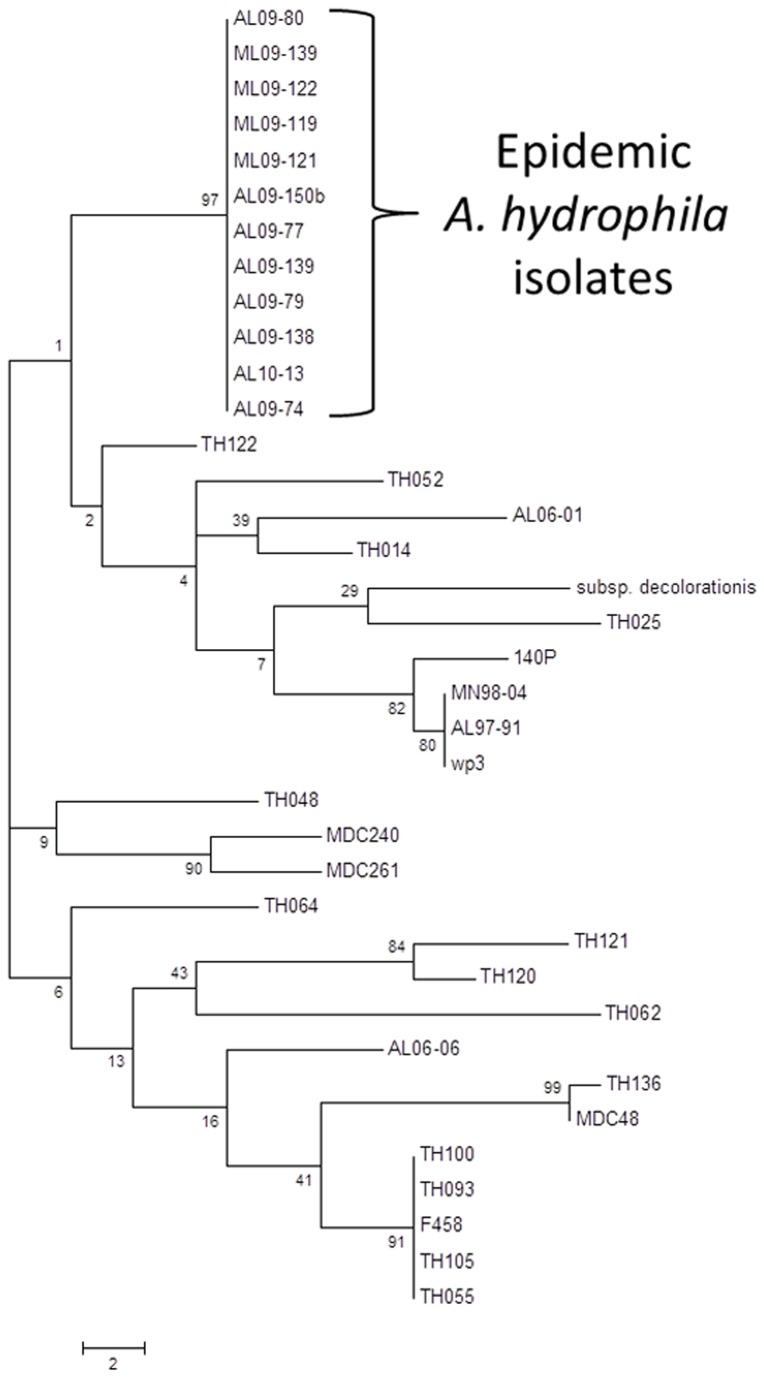
Evolutionary relationships of 37 *A. hydrophila* taxa based on *gyrB* gene sequences (out of a larger dataset of 107 *A. hydrophila* *gyrB* sequences). The evolutionary history was inferred using the Maximum Parsimony method. Tree #1 out of 67 most parsimonious trees (length = 218) is shown. The percentage of replicate trees in which the associated taxa clustered together in the bootstrap test (1000 replicates) are shown next to the branches. The tree is drawn to scale, with branch lengths calculated using the average pathway method and are in the units of the number of changes over the whole sequence.

Phylogenetic analysis based on 16S rRNA gene sequences of epidemic *A. hydrophila* isolates demonstrated they are 100% identical to previously reported *A. hydrophila* strains (data not shown). Phylogenetic analysis based on *gyrB* gene sequences of representative *A. hydrophila* isolates demonstrated sufficient resolution to separate VAh and RAh isolates. A maximum parsimony (MP) tree generated from the alignment of VAh and RAh *gyrB* nucleotide sequences revealed that all of the VAh strains consistently grouped together as a single clade with strong bootstrap support (Figure 1). Although the VAh isolates formed a coherent clade based on *gyrB* gene sequences, there were not sufficient phylogenetically informative nucleotide positions within the *gyrB* gene sequence to develop a VAh-specific primer set. 

### *A. hydrophila* genome sequencing, assembly and annotation

Summary statistics for each of the *A. hydrophila* genome sequences and their assemblies are provided in Table 1. The average number of contigs obtained per genome was 114. The nucleotide sequences for strain ML09-119 contigs are provided in the Dataset S1. After trimming, the quality Illumina sequence reads totaled 9510.8 Mb, with an average coverage of >160-fold per genome. The 454 pyrosequencing of an induced prophage from strain ML09-119 (see below) provided a total of 96,598 high-quality sequences with an average read length of 268 bp. The average genome size of the VAh and RAh isolates were 5.0 Mb and 4.8 Mb, respectively. The %G+C content of the 11 strains ranged from 60.5% to 61.5% (Table 1) which was consistent with the previously reported %G+C content of 61.5 % for *A. hydrophila* ATCC 7966 [19]. Protein sequences from all of the predicted open reading frames (ORFs) of *A. hydrophila* VAh strain ML09-119 and all RAh strain genomes are listed in Dataset S2.

### Identification of unique genomic regions associated with VAh isolates

The VAh strain ML09-119, originally cultured from the kidney tissue of a diseased channel catfish demonstrating characteristic MAS symptoms, has been typed as an *A. hydrophila* strain by biochemistry and 16S rRNA gene sequencing and is highly virulent in channel catfish, and was used as a type strain for all further analyses in this study. We found that the ML09-119 genome contains 55 unique regions (Dataset S3) that are missing in all five RAh isolates (AL06-01, AL06-06, AL97-91, MN98-04 and TN97-08) and *A. hydrophila* ATCC 7966. These 55 unique regions are also present in all five of the other sequenced VAh isolates (AL09-79, AL10-121, ML09-121, ML09-122, and PB10-118). These epidemic-associated regions contain 336,469 bp, accounting for 6.7% of the ML09-119 genome. A total of 313 ORFs are encoded by these unique regions (Datasets S4 and S5). Region C2R1 is the smallest region with one predicted ORF, whereas C15R7 is the largest region (33,402 bp) predicted to encode 36 different proteins. More than 252,453 bp of these unique sequences are part of 16 predicted genomic islands (GIs; see below for detailed description of these GIs). About 51% (160 out of 313) of the VAh-associated genes are predicted to encode proteins with unknown functions (Dataset S6). The average %G+C content of these unique regions is 47.0%, whereas the %G+C content of the ML09-119 genome is 60.9%. The %G+C content bias of the VAh-associated regions supports the hypothesis that novel genomic segments were acquired through LGT. 

### Determining the Pan and Core-genome of *A. hydrophila*

**Figure 2:**
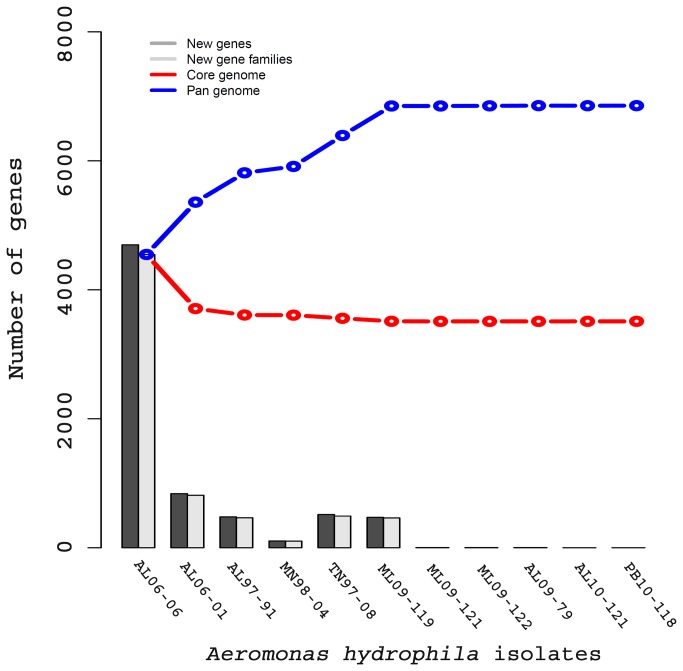
Pan and core-genome plot of 11 different *A. hydrophila* isolates. The red and blue lines indicated the number of genes within the core and pan-genomes, respectively. The *A. hydrophila* core genome contained 3,511 core genes whereas the *A. hydrophila* pan-genome contained 6,856 genes. Note that the addition of other VAh strains after *A. hydrophila* ML09-119 did not significantly increase the number of new gene families, which was in agreement with the highly clonal nature of VAh strains.

A total of 6,856 pan-gene families comprising full sets of non-orthologous genes families were found within the genome of the 11 *A. hydrophila* isolates. We found 3,511 conserved core gene families within these 11 *A. hydrophila* genomes. Based on these estimates the number of pan-genes is approximately twice the number of core-genes in *A. hydrophila*. Considering the 4,765 average number of gene families present in each of the *A. hydrophila* isolates sequenced in this study, it was observed that 74% of the predicted genes were core genes that are shared among all of the *A. hydrophila* isolates. From a plot of pan- and core-genomes it was observed that the number of genes in the pan-genomes reached its maximum among the VAh strains (Figure 2). There was a negligible increase in the number of new gene families among the VAh genomes, supporting the conclusion that the six epidemic *A. hydrophila* genomes are highly similar.

### Pairwise proteome comparison of *A. hydrophila* genomes

**Figure 3:**
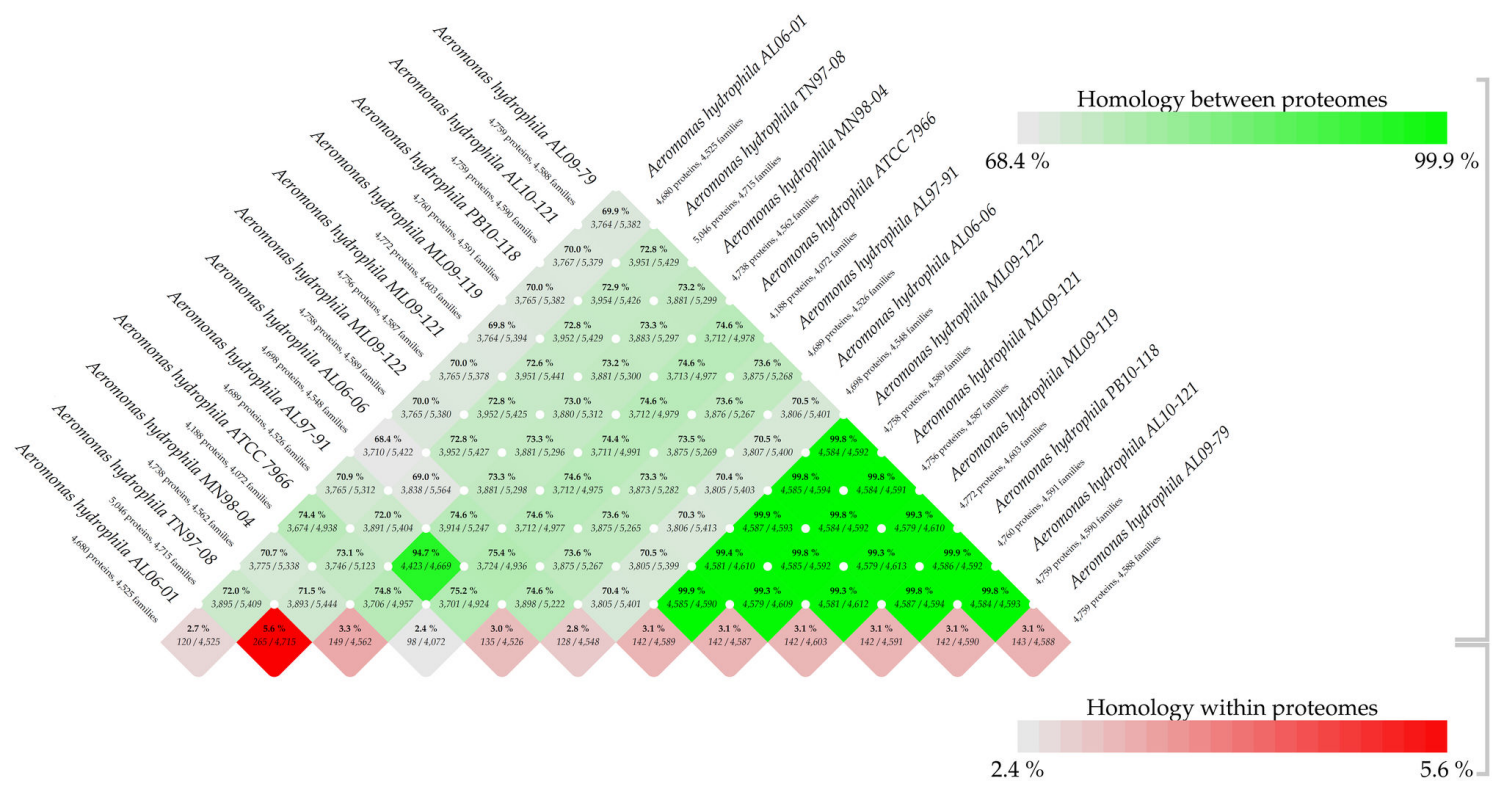
BLAST MATRIX of 12 different *A. hydrophila* isolates. The proteomes of each of the *A. hydrophila* strains were compared using all-against-all BLASTp according to the methods described previously [29]. This matrix showed the output from pairwise comparison of conserved protein families of each of the isolates to each other. The green color represents the % homology between proteomes and the red color represents % homology within proteomes. This matrix showed that all the epidemic *A. hydrophila* isolates are similar to each other but substantially different to reference isolates. All of the reference isolates, except for MN98-04 and AL97-91 that were highly similar to each other, possessed substantial amount of diversity in their protein families.

A pairwise BLAST Matrix was generated to determine the similarity in each of the conserved protein families present within the *A. hydrophila* genomes. The proteome comparison revealed that the average protein family similarity between any two *A. hydrophila* genomes ranges from 68.4-99.9% while the intra-proteome homology among protein families within each isolates is less than 5.6% (Figure 3). The pairwise comparison of proteomes showed that the six VAh strains share a very high degree of homology (>99%) (Figure 3, highlighted with triangle). In contrast, the pairwise proteome comparison between the VAh and RAh strains and among RAh strains including *A. hydrophila* ATCC 7966 revealed a range from 68.4 to 94.7% homology. These results demonstrated that VAh strains are genomically distinct from RAh strains and that there is a highly coherent VAh genome. The BLAST matrix results also indicated that RAh proteomes are diverse, with an average 72.91% sequence identity. One exception was the 95% sequence identity between the AL97-91 and MN98-04 proteomes. Since these two strains had been isolated from Tilapia, this may reflect their isolation from a common host fish. These results suggest there was significant diversity among RAh strains sampled in this study, especially in contrast to VAh strain genomic homogeneity.

### Novel O-antigen biosynthesis gene cluster in VAh isolates

**Figure 4:**
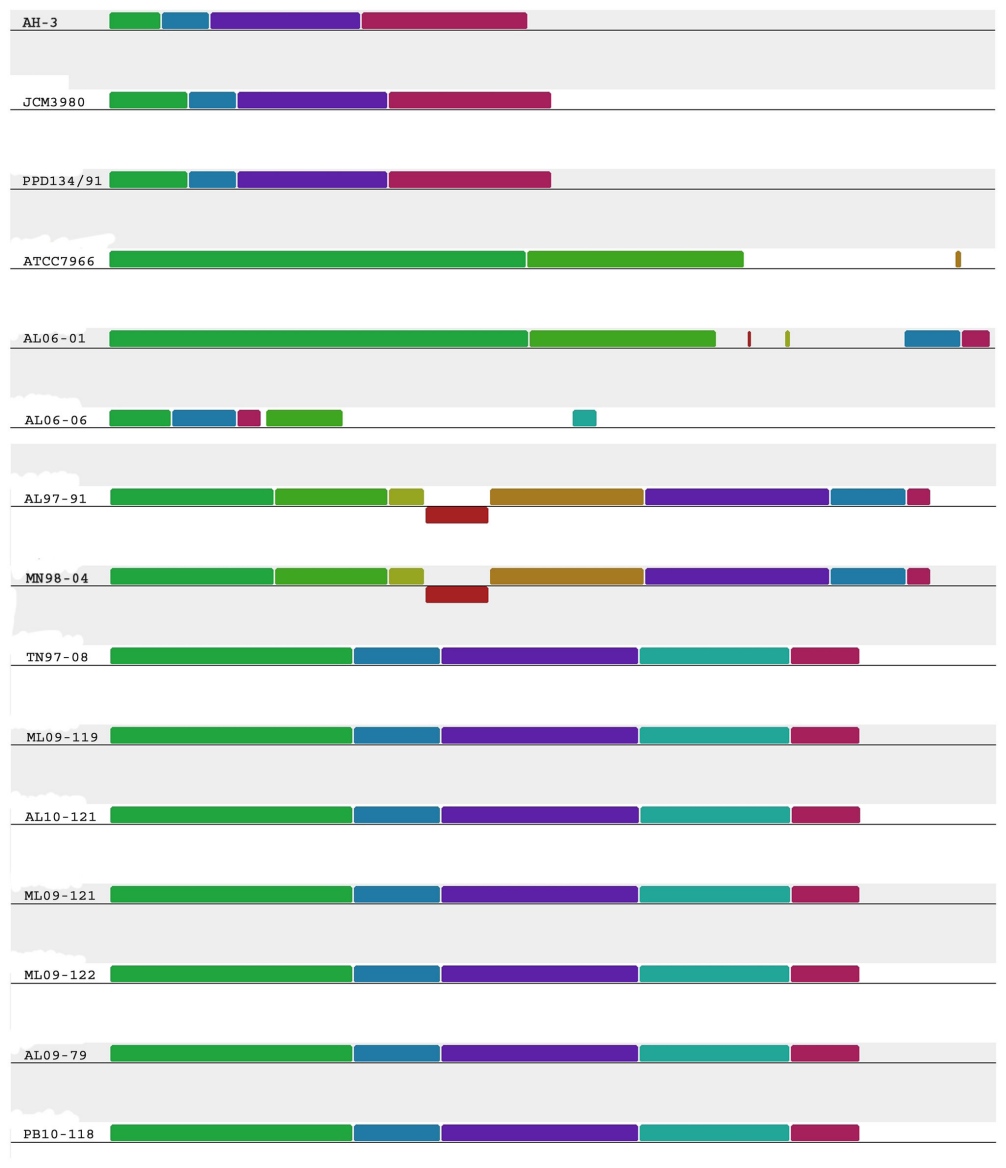
Mauve Alignment of the O-antigen cluster from 16 different *A. hydrophila* isolates. Segments with a similar color indicate homologous regions. The alignment is on scale based on the size of the O-antigen cluster of *A. hydrophila* ATCC 7966 which is 44 kb in length. All of the EAh strains including RAh strain TN97-08 shared the ML09-119-type O-antigen biosynthesis gene cluster.

The significant role of O-antigen, the most variable surface structure in terms of its composition, in bacterial virulence [37] prompted us to analyze the O-antigen biosynthesis gene cluster of *A. hydrophila* isolates. We found that all of the VAh isolates and one RAh isolate TN97-08 shared a 26.5 kb novel O-antigen biosynthesis gene cluster predicted to encode 25 different ORFs (Tables 2 and 3, Figure 4). Though the proteomic comparison of the TN97-08 and VAh isolates showed about 73% similarities (Figure 3), the sharing of an entire O-antigen biosynthesis cluster suggests possible LGT events. None of the O-antigen biosynthesis gene clusters of RAh isolates, except for strain TN98-04, shared homology with that of the VAh isolates and five of the RAh isolates possess four unique O-antigen biosynthesis clusters (Figure 4). Among the five RAh isolates, only AL97-91 and MN98-04 isolates shared homology in their O-antigen cluster (Figure 4), which is in agreement with their overall proteomic homology (Figure 3). The O-antigen biosynthesis cluster of 11 sequenced *A. hydrophila* isolates are quite different than the previously published *A. hydrophila* ATCC 7966 [19], PPD134/91 [38], JCM3980 [38] and AH-3 [39] O-antigen biosynthesis clusters (Figure 4). 

**Table 2:** Summary of ORFs encoded by the O-antigen biosynthesis gene cluster of VAh strain ML09-119.

ORF ID	Nucleotide positions in Accession no. KC999973	Predicted Function	Gene	Nearest Neighbor	% Identity	Accession number
ORFu	1.672	lipoprotein YmcC	*ymcC*	*Edwardsiella tarda* ATCC 23685	43	ZP_06715179.1
ORF1	1114.1842	O-antigen length determinant protein	*wzzA*	*Ferrimonas balearica* DSM 9799	59	YP_003912353.1
ORF2	2160..4784	periplasmic protein involved in polysaccharide export	*wza*	*Marinomonas* *sp.* MWYL1	45	YP_001339663.1
ORF3	4930.6012	O-antigen chain length determinant protein	*wzzB*	*Aeromonas veronii* B565	80	YP_004393425.1
ORF4	6067.6606	dTDP-4-dehydrorhamnose 3,5-epimerase	*rmlC*	*Halomonas* *sp.* TD01	58	ZP_08635572.1
ORF5	6620.7561	glucose-1-phosphate thymidylyltransferase	*rmlA*	*Lutiella nitroferrum* 2002	61	ZP_03699710.1
ORF6	7561.8445	rmlD gene product	*rmlD*	*gamma proteobacterium* HdN1	54	YP_003809930.1
ORF7	8432.9493	undecaprenyl-phosphate N-acetylglucosaminyl 1-phosphate transferase	*wecA*	*Photobacterium damselae* subsp. *damselae* CIP 102761	57	ZP_06154788.1
ORF8	9544.10776	phosphomannomutase	*manB*	*Vibrio nigripulchritudo* ATCC 27043	69	ZP_08734182.1
ORF9	10965.11717	Colanic acid biosynthesis glycosyl transferase	*wcaE*	*Shigella dysenteriae* 1012	67	ZP_03065870.1
ORF10	11717.13018	mannose-1-phosphate guanylyltransferase	*manC*	*Photobacterium profundum* 3TCK	71	ZP_01218698.1
ORF11	13128.13610	GDP-mannose mannosyl hydrolase	*gmm*	*Tolumonas auensis* DSM 9187	63	YP_002893236.1
ORF12	13594.14574	GDP-fucose synthetase	*fcl*	*Yersinia pestis* KIM 10	84	NP_668408.1
ORF13	14578.15684	GDP-mannose 4,6-dehydratase	*gmd*	*Vibrio angustum* S14	86	ZP_01235027.1
ORF14	15705.16940	group 1 glycosyl transferase	*wbxH*	*Pectobacterium carotovorum* subsp. *carotovorum* WPP14	49	ZP_03830724.1
ORF15	16937.17986	group 1 glycosyl transferase	*wbxH*	*Pectobacterium carotovorum* subsp. *carotovorum* PC1	52	YP_003016893
ORF16	17986.19167	glycosyl transferase group 1	*wbxI*	*Methylobacter tundripaludum* SV96	52	ZP_08780763.1
ORF17	19164.19655	acetyltransferase	*wcaF*	*Methylobacter tundripaludum* SV96	59	ZP_08780764.1
ORF18	19648.20826	O-antigen polymerase	*wzyE*	*Bacteroides* *sp.* 2_1_7	29	ZP_05287114.1
ORF19	20877.21968	group 1 glycosyl transferase protein	*wbxU*	*Dysgonomonas gadei* ATCC BAA-286	42	ZP_08475479.1
ORF20	22444.23694	O-antigen flippase	*wzxB*	*Shewanella baltica* OS625	81	EHC06312.1
ORF21	23691.24794	aminotransferase	*fdtB*	*Shewanella baltica* OS195	81	YP_001555451.1
ORF22	24796.25233	dTDP-D-Fucp3N acetyltransferase	*fdtC*	*Shewanella baltica* OS195	85	YP_001555452.1
ORF23	25235.25657	dTDP-6-deoxy-3,4-keto-hexulose isomerase	*fdtA*	*Shewanella putrefaciens* 200	72	ADV52549.1
ORF24	25668.26534	glucose-1-phosphate thymidylyltransferase	*rmlA*	*Shewanella putrefaciens* 200	79	NP_718742
ORF25	26531.27619	dTDP-glucose-4-6-dehydratase	*rmlB*	*Aeromonas hydrophila*	93	AAM22544.1
ORFd	28234.31383	AcrB protein	*acrB*	*Aeromonas hydrophila* subsp. *hydrophila* ATCC 7966	99	YP_857414.1

**Table 3:** Summary of ORFs encoded by the O-antigen biosynthesis gene cluster of RAh strain TN97-08.

ORF ID	Nucleotide positions in Accession no. KC999968	Predicted Function	Gene	Nearest Neighbor	% Identity	Accession number
ORFu	1.672	lipoprotein YmcC	*ymcC*	*Edwardsiella tarda* ATCC 23685	43	ZP_06715179.1
ORF1	1114.1842	O-antigen length determinant protein	*wzz*	*Ferrimonas balearica* DSM 9799	59	YP_003912353
ORF2	2160..4784	periplasmic protein involved in polysaccharide export	*otnA*	*Marinomonas* *sp.* MWYL1	45	YP_001339663
ORF3	4930.6012	O-antigen chain length determinant protein	*wzz*	*Aeromonas veronii* B565	80	YP_004393425
ORF4	6067.6606	dTDP-4-dehydrorhamnose 3,5-epimerase	*rmlC*	*Halomonas* *sp.* TD01	58	ZP_08635572
ORF5	6620.7561	glucose-1-phosphate thymidylyltransferase	*rmlA*	*Lutiella nitroferrum* 2002	61	YP_005093462
ORF6	7561.8445	rmlD gene product	*rmlD*	*gamma proteobacterium* HdN1	54	YP_003809930
ORF7	8432.9493	undecaprenyl-phosphate N-acetylglucosaminyl 1-phosphate transferase	*wecA*	*Photobacterium damselae* subsp. *damselae* CIP 102761	57	ZP_06154788
ORF8	9544.10776	phosphomannomutase	*manB*	*Vibrio nigripulchritudo* ATCC 27043	69	YP_005021705
ORF9	10965.11717	Colanic acid biosynthesis glycosyl transferase	*wcaE*	*Shigella dysenteriae* 1012	67	ZP_03065870
ORF10	11717.13018	mannose-1-phosphate guanylyltransferase	*manC*	*Photobacterium profundum* 3TCK	71	ZP_01218698
ORF11	13128.13610	GDP-mannose mannosyl hydrolase	*gmm*	*Tolumonas auensis* DSM 9187	63	YP_002893236
ORF12	13594.14574	GDP-fucose synthetase	*fcl*	*Yersinia pestis* KIM 10	84	NP_668408
ORF13	14578.15684	GDP-mannose 4,6-dehydratase	*gmd*	*Vibrio angustum* S14	86	ZP_01235027
ORF14	15705.16940	group 1 glycosyl transferase	*wbxH*	*Pectobacterium carotovorum* subsp. *carotovorum* WPP14	49	ZP_03830724
ORF15	16937.17986	group 1 glycosyl transferase	*wbxH*	*Pectobacterium carotovorum* subsp. *carotovorum* PC1	52	YP_003016893
ORF16	17986.19167	glycosyl transferase group 1	*wbxI*	*Methylobacter tundripaludum* SV96	52	ZP_08780763
ORF17	19164.19655	acetyltransferase	*wcaF*	*Methylobacter tundripaludum* SV96	59	ZP_08780764
ORF18	19648.20826	O-antigen polymerase	*wzyE*	*Bacteroides* *sp.* 2_1_7	29	ZP_05287114
ORF19	20877.21968	group 1 glycosyl transferase protein	*wdaN*	*Dysgonomonas gadei* ATCC BAA-286	42	ZP_08475479
ORF20	22444.23694	O-antigen flippase	*wzxB*	*Shewanella baltica* OS625	81	EHC06312
ORF21	23691.24794	aminotransferase	*fdtB*	*Shewanella baltica* OS195	81	YP_001555451
ORF22	24796.25233	dTDP-D-Fucp3N acetyltransferase	*fdtC*	*Shewanella baltica* OS195	85	YP_001555452
ORF23	25235.25657	dTDP-6-deoxy-3,4-keto-hexulose isomerase	*fdtA*	*Shewanella putrefaciens* 200	72	ADV52549
ORF24	25668.26534	glucose-1-phosphate thymidylyltransferase	*rmlA*	*Shewanella putrefaciens* 200	79	ADV52548
ORF25	26531.27619	dTDP-glucose-4-6-dehydratase	*rmlB*	*Aeromonas hydrophila*	93	AAM22544
ORFd	28234.31383	AcrB protein	*acrB*	*Aeromonas hydrophila* subsp. *hydrophila* ATCC 7966	99	YP_857414.1

The analysis of gene content within the VAh O-antigen cluster suggests that VAh strains express a previously uncharacterized O-antigen among *A. hydrophila* strains. The comparison of the VAh O-antigen biosynthesis cluster with that of strains PPD134/91 and AH-3 revealed that the VAh O-antigen gene cluster (26.5 kb) is larger than the 19 kb clusters contained in both PPD134/91 and AH-3. The analysis of the VAh O-antigen gene cluster revealed 25 ORFs and the annotation of each ORF demonstrated that this O-antigen gene cluster contains predicted gene products required for the synthesis of activated nucleotide sugars, transport of those sugars to the growing O-antigen chain, and the processing of the O-antigen (Table 2). The VAh O-antigen biosynthesis cluster contains genes putatively required for the synthesis of nucleotide activated sugars D-rhamnose, D-mannose, GDP-L-Fucose and 3-acetamido-3, 6-dideoxy-d-galactose (D-Fuc*p*3NAc). The *rmlA*, *rmlB*, *rmlC* and *rmlD* genes are usually clustered together [40] and products of those genes are required for the synthesis of dTDP-rhamnose. Each of the VAh and RAh strains possess *rmlA, rmlB, rmlC and rmlD* genes in their O-antigen gene cluster. Though each of the unique O-antigen cluster types described in this study contain genes required for D-rhamnose biosynthesis, the homology and organization of those genes varied substantially (Tables 2-6). Those genes are also present within the antigen clusters of *A. hydrophila* ATCC 7966, PPD134/91, JCM3980 and AH-3. These collective observations indicate that D-rhamnose is the most common sugar component present within the O-antigen of *A. hydrophila*. The presence of all five of the genes required for D-Fuc*p*3NAc synthesis in the VAh O-antigen cluster suggests that this sugar is also a major component of the VAh O-antigen. In contrast, the O-antigen clusters of all RAh and previously sequenced *A. hydrophila* strains do not contain the *fdtA*, *fdtB* or *fdtC* genes required for D-Fucp3NAc synthesis. 

**Table 4:** Summary of ORFs encoded by the O-antigen biosynthesis gene cluster of RAh strain AL06-01.

ORF ID	Nucleotide positions in Accession no. KC999970	Predicted function	Gene	Nearest Neighbor	%Identity	Accession Number
ORFu	1..429	low molecular weight protein-tyrosine-phosphatase	*ppt*	*Aeromonas hydrophila* subsp. *hydrophila* ATCC 7966	99	YP_857382
ORF1	665..1780	capsular polysaccharide transport protein	*wza*	*Aeromonas hydrophila* subsp. *hydrophila* ATCC 7966	99	YP_857383
ORF2	2116..3282	UDP-glucose 6-dehydrogenase	*wbpO*	*Aeromonas hydrophila* subsp. *hydrophila* ATCC 7966	99	YP_857384
ORF3	3295..4308	nucleotide sugar epimerase	*wcaG*	*Aeromonas hydrophila* subsp. *hydrophila* ATCC 7966	99	YP_857385
ORF4	4305..5459	O-antigen polymerase	*wzyE*	*Vibrio alginolyticus* 40B	32	ZP_06180045
ORF5	5549..6343	glycosyl transferase family protein	*wbxJ*	*Aeromonas hydrophila* subsp. *hydrophila* ATCC 7966	86	YP_857387
ORF6	6345..7166	glycosyl transferase family protein	*wbxJ*	*Aeromonas hydrophila* subsp. *hydrophila* ATCC 7966	98	YP_857388
ORF7	7170..8264	glycoside hydrolase family protein	*wbxV*	*Aeromonas hydrophila* subsp. *hydrophila* ATCC 7966	99	YP_857389
ORF8	8327..9541	polysaccharide biosynthesis protein	*csaB*	*Aeromonas hydrophila* subsp. *hydrophila* ATCC 7966	100	YP_857390
ORF9	9543..10658	NAD(P) transhydrogenase subunit alpha	*adl*	*Aeromonas hydrophila* subsp. *hydrophila* ATCC 7966	100	YP_857391
ORF10	10651..11673	RimK-like protein	*rimK*	*Aeromonas hydrophila* subsp. *hydrophila* ATCC 7966	100	YP_857392
ORF11	11732..12811	UDP-phosphate alpha-N-acetylglucosaminyl 1-phosphatetransferase	*wecA*	*Aeromonas hydrophila* subsp. *hydrophila* ATCC 7966	100	YP_857393
ORF12	13296..15281	protein WbgZ	*wbgZ*	*Aeromonas hydrophila* subsp. *hydrophila* ATCC 7966	98	YP_857395
ORF13	15278..16156	glycoside hydrolase family protein	*wbpI*	*Aeromonas hydrophila* subsp. *hydrophila* ATCC 7966	92	YP_857396
ORF14	16303..17259	UDP-glucose 4-epimerase	*galE*	*Aeromonas hydrophila* subsp. *hydrophila* ATCC 7966	91	YP_857397
ORF15	17259..18068	glycosyl transferase family protein	*wbxW*	*Aeromonas salmonicida* subsp. *salmonicida* A449	65	YP_001141293
ORF16	18059..19144	glycosyltransferase	*wbxX*	*Enterobacter aerogenes* KCTC 2190	53	YP_004594810
ORF17	19141..20250	UDP-N-acetylglucosamine 2-epimerase	*wecB*	*Aeromonas salmonicida* subsp. *salmonicida* A449	80	YP_001141289
ORF18	20262..21338	group 1 glycosyl transferase	*wbxY*	*Enterobacter aerogenes* KCTC 2190	43	YP_004594808
ORF19	21325..24027	family 2 glycosyl transferase	*wbxZ*	*Enterobacter aerogenes* KCTC 2190	47	YP_003444096
ORF20	24040..25344	ABC transporter ATP-binding protein	*rfbE*	*Enterobacter aerogenes* KCTC 2190	64	YP_004594806
ORF21	25334..26143	ABC-2 type transporter	*rfbD*	*Pseudomonas chlororaphis* O6	60	ZP_10171957
ORF22	26145..26684	dTDP-4-dehydrorhamnose 3,5-epimerase	*rmlC*	*Aeromonas caviae* Ae398	78	ZP_08521430
ORF23	26747..27625	glucose-1-phosphate thymidylyltransferase 1	*rmlA*	*Aeromonas veronii* B565	92	YP_001141271
ORF24	27737..28624	dTDP-4-dehydrorhamnose reductase	*rmlD*	*Aeromonas hydrophila* subsp. *hydrophila* ATCC 7966	95	YP_857411
ORF25	28624..29709	putative dTDP-glucose-4-6-dehydratase	*rmlB*	*Aeromonas hydrophila*	96	AAM22544
ORFd	30397..31806	outer membrane protein OprM	*oprM*	*Aeromonas hydrophila* subsp. *hydrophila* ATCC 7966	97	YP_857413

**Table 5:** Summary of ORFs encoded by the O-antigen biosynthesis gene cluster of RAh strain AL06-06.

ORF ID	Nucleotide positions in Accession no. KC999971	Predicted function	Gene	Nearest Neighbor	%Identity	Accession Number
ORFu	1..1719	lipid A core - O-antigen ligase	*waaL*	*Aeromonas hydrophila* subsp. *hydrophila* ATCC 7966	97	YP_857377
ORF1	1814..2875	O-antigen chain length determinant protein	*wzzC*	*Aeromonas caviae* Ae398	69	ZP_08521419
ORF2	2948..3487	dTDP-4-dehydrorhamnose 3,5-epimerase	*rmlC*	*Halomonas* *sp.* TD01	57	ZP_08635572
ORF3	3592..4479	glucose-1-phosphate thymidylyltransferase 1	*rmlA*	*Aeromonas veronii* B565	92	YP_004392190
ORF4	4591..5478	dTDP-4-dehydrorhamnose reductase	*rmlD*	*Aeromonas hydrophila* subsp. *hydrophila* ATCC 7966	97	YP_857411
ORF5	5478..6566	dTDP-glucose-4, 6-dehydratase	*rmlB*	*Aeromonas hydrophila*	98	AAM22544
ORF6	6761..8722	epimerase/dehydratase family WbfY-like protein	*wbgZ*	*Aeromonas caviae* Ae398	96	ZP_08521420
ORF7	8781..9335	lipid carrier : UDP-N-acetylgalactosaminyltransferase	*wbtB*	*Aeromonas veronii* B565	96	YP_004393428
ORF8	9338..10078	UDP-glucose 4-epimerase	*galE*	*Vibrio metschnikovii* CIP 69.14	77	ZP_05883342
ORF9	10301..11422	glycosyl transferase, group 1 family protein	*wbxK*	*Shewanella oneidensis* MR-1	59	NP_718730
ORF10	11419..13353	asparagine synthetase, glutamine-hydrolyzing	*asnD*	*Shewanella oneidensis* MR-1	74	NP_718731
ORF11	13380..14491	glycosyl transferase, group 1	*wbxL*	*Vibrio ichthyoenteri* ATCC 700023	46	ZP_08744363
ORF12	14505..15575	group 1 glycosyl transferase	*wbxM*	*Achromobacter piechaudii* ATCC 43553	35	ZP_06689986
ORF13	15572..16861	virulence factor MVIN family protein	*mviN*	*Burkholderia ubonensis* Bu	39	ZP_02383242
ORF14	16858..18057	Cna B domain-containing protein	*cnaB*	*Flavobacterium* sp. F52	33	ZP_10479786
ORF15	18286..19314	UDP-GlcNAc 4-epimerase	*wbgU*	*Shewanella oneidensis* MR-1	81	NP_718745
ORF16	19417..20697	UDP-glucose dehydrogenase	*wbpO*	*Vibrio vulnificus* MO6-24/O	88	YP_004190001
ORF17	21479..21766	dTDP-D-glucose-4,6-dehydratase	*rmlB*	*Aeromonas hydrophila*	90	AAM74474
ORFd	22510..23925	outer membrane protein OprM	*oprM*	*Aeromonas hydrophila*	99	AAM22559

The VAh O-antigen gene cluster was predicted to contain five different genes, namely *gmd, fcl, gmm, manC*, and *manB* required for the synthesis of GDP-mannose and GDP-L-fucose from fructose-6-phosphate (Table 2). ManA, ManB, and ManC are required for the synthesis of GDP-mannose from fructose-6-phosphate. Typically, the genes *manB* and *manC* are located within the O-antigen cluster, whereas *manA* is found outside the O-antigen cluster elsewhere within the genome [38,39,41] and this was also observed for the VAh O-antigen cluster. The genes *gmd* and *fcl* encode GDP-mannose 4, 6-dehydratase and GDP-L-fucose synthetase, respectively, which synthesize GDP-L-fucose using GDP-mannose as a precursor. None of the previously sequenced O-antigen biosynthesis gene clusters of *A. hydrophila* contained *gmd* or *fcl* genes. The *fdtA*, *fdtC* and *fdtB* genes are predicted to encode the enzymes dTDP-6-deoxy-3,4-keto-hexulose isomerase (FdtA), dTDP-D-Fucp3N acetyltransferase (FdtC), and aminotransferase (FdtB), respectively, required for the synthesis of the dTDP-sugar 3-acetamido-3,6-dideoxy-D-galactose (dTDP-D-Fucp3NAc) [42], an activated nucleotide sugar that could be incorporated into the VAh O-antigen. In addition to these enzymes, D-glucose-1-phosphate thymidyltransferase (RmlA) and dTDP-D-glucose-4,6-dehydratase (RmlB) encoded by the *rmlA* and *rmlB* genes, respectively, are predicted within the VAh O-antigen cluster and are required for the biosynthesis of the nucleotide sugar dTDP-D-Fucp3NAc [42]. 

The VAh O-antigen cluster contained five different glycosyltransferase genes and one acetyltranferase gene (Table 2). A series of glycosyltransferases work consecutively to assemble the nucleotide sugar repeat on the membrane lipid undecaprenol pyrophosphate (Und-PP). The VAh O-antigen gene clusters were predicted to contain the *wecA* gene that encodes a undecaprenyl-phosphate alpha-N-acetylglucosaminyl 1-phosphate transferase required for the transfer of the GlcNAc-1-phosphate moiety from UDP-GlcNAc onto the carrier lipid undecaprenyl phosphate. The single polysaccharide repeat bound to Und-PP is flipped to the periplasmic side which is catalyzed by O-antigen flippase [43] and polymerized by the Wzy-dependent pathway [44]. The VAh strains were also found to possess an O-antigen flippase (*wzxB*) and O-antigen polymerase (*wzyE*) within their O-antigen gene cluster. These findings suggest the presence of smooth LPS on each of the VAh strains. 

### Comparative analysis of O-antigen biosynthesis gene clusters in RAh strains

**Figure 5:**
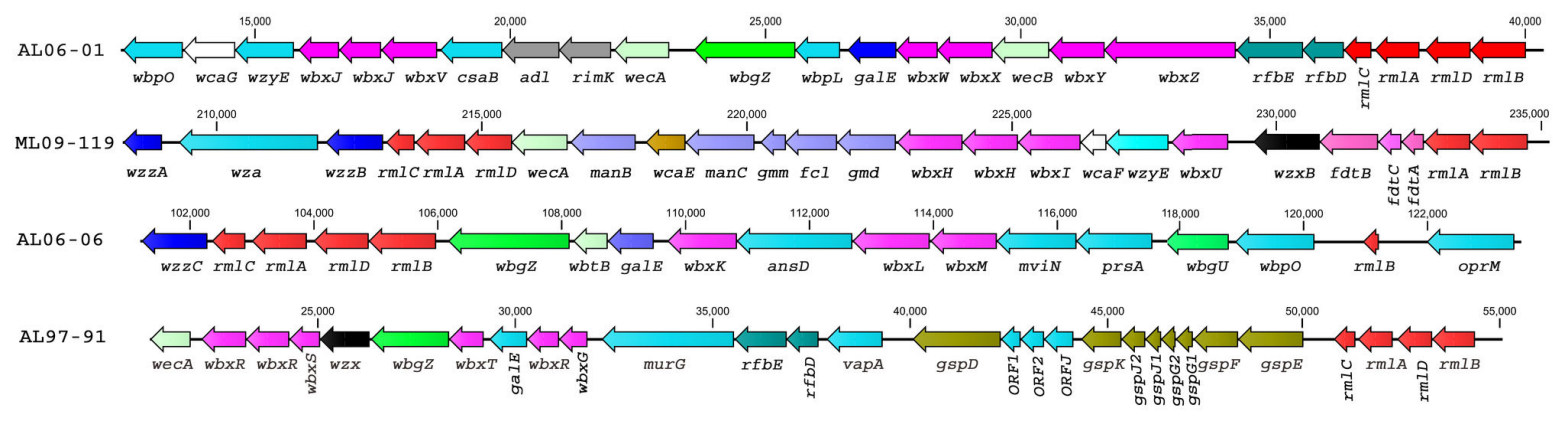
Schematic organization of the four different types of O-antigen biosynthesis gene cluster present within the genome of the 11 *A. hydrophila* isolates sequenced in this study. All of the genes on the cluster are transcribed in the same direction. All VAh strains along with RAh strain TN97-08 shared the ML09-119-type O-antigen biosynthesis gene cluster. This cluster encodes proteins predicted to be involved in the biosynthesis of the nucleotide sugars D-rhamnose, D-mannose, D-Fucose, and 3-acetamido-3, 6-dideoxy-d-galactose (D-Fuc*p*3NAc). The AL97-91-type cluster (that was also shared with MN98-04) encodes genes predicted to be required for S-layer biosynthesis and transport in addition to O-antigen biosynthesis. Genes that encode conserved proteins with similar functions are marked with the same color. The number displayed next to the maps indicates the nucleotide positions on the respective contig from each strain. The designation of each of the genes presented on the schematic map of the AL06-01, ML09-119, AL06-06 and AL97-91 O-antigen clusters are found in Tables 2, 4, 5 and 6, respectively.

The predicted O-antigen clusters in the RAh strains AL06-01 and AL06-06 are unique. The O-antigen biosynthesis gene cluster of AL06-01 and AL06-06 are 29 kb and 18.86 kb in length that encode 25 and 17 ORFs, respectively. The gene content and organization of O-antigen clusters of AL06-01 and AL06-06 varied substantially with those of the other VAh and RAh strains used in this study (Tables 2-6 and Figure 5). The AL06-01 strain contains all 4 genes (*rmlA*, *rmlB*, *rmlC* and *rmlD*) required for the biosynthesis of dTDP-rhamnose from glucose-1-phosphate. It also contains gene that encode UDP-glucose 6-dehydrogenase required for the biosynthesis of UDP-D-glucuronic acid from UDP-glucose. These findings suggest the presence of D-rhamnose and D-glucuronic acid on the O-antigen of strain AL06-01. The unique O-antigen cluster of RAh strains AL06-01 and AL06-06 do not contain any genes required for the synthesis of D-mannose or D-L-fucose (Tables 4 and 5). Instead, the AL06-06 O-antigen cluster is predicted to encode UDP-N-acetyl-D-galactosamine dehydrogenase and an epimerase/dehydratase family WbfY-like protein. Those two enzymes are required for the biosynthesis of UDP-GalNAcA which is a common O-antigen sugar for many Gram-negative bacteria.

**Table 6:** Summary of ORFs encoded by the O-antigen biosynthesis gene cluster of RAh strain AL97-91.

ORF ID	Nucleotide positions in Accession no. KC999966	Predicted function	Gene	Nearest Neighbor	% Identity	Accession Number
ORFu	1.1710	lipid A core - O-antigen ligase	*waaL*	*Aeromonas hydrophila* subsp. *hydrophila* ATCC 7966	97	YP_857377
ORF1	1715..2740	UDP-glucose lipid carrier transferase	*wecA*	*Aeromonas hydrophila* subsp. *hydrophila* ATCC 7966	73	ABX39510
ORF2	3008.4144	glycosyltransferase	*wbxR*	*Aeromonas salmonicida* subsp. *salmonicida* A449	75	YP_001141302
ORF3	4141.5235	glycosyltransferase, group 2 family protein	*wbxR*	*Aeromonas salmonicida* subsp. *salmonicida* A449	70	YP_001141301
ORF4	5237.6016	glycosyltransferase, group 2 family protein	*wbxS*	*Escherichia coli*	48	ACH97156
ORF5	6013.7269	integral membrane protein AefA/O-antigen flippase	*wzx*	*Salmonella bongori* NCTC 12419	70	YP_004730750
ORF6	7300.9282	epimerase/dehydratase family WbfY-like protein	*wbgZ*	*Aeromonas hydrophila* subsp. *hydrophila* ATCC 7966	94	YP_857395
ORF7	9279.10157	glycosyl transferase, group 4 family protein	*wbxT*	*Aeromonas hydrophila* subsp. *hydrophila* ATCC 7966	91	YP_857396
ORF8	10304.11260	UDP-glucose 4-epimerase	*galE*	*Aeromonas hydrophila* subsp. *hydrophila* ATCC 7966	92	YP_857397
ORF9	11260.12066	Glycosyltransferase, family 2	*wbxR*	*Aeromonas salmonicida* subsp. *salmonicida* A449	65	YP_001141293
ORF10	12068.12787	acyltransferase family protein	*wbxG*	*Flavobacteria bacterium* BAL38	30	ZP_01733088
ORF11	13147.16497	putative N-acetyl glucosaminyl transferase	*murG*	*Serratia odorifera* 4Rx13	50	ZP_06189367
ORF12	16515.17831	transporter	*rfbE*	*Serratia odorifera* 4Rx13	73	ZP_06189366
ORF13	17821.18639	ABC-2 type transporter	*rfbD*	*Thermosinus carboxydivorans* Nor1	65	ZP_01665322
ORF14	18841.20259	surface layer protein	*vapA*	*Aeromonas hydrophila*	100	ACV89427
ORF15	21019.23250	S-protein secretion D	*gspD*	*Aeromonas hydrophila*	100	AAA79322
ORF16	23254.23754	ORF2	ORF2	*Aeromonas hydrophila*	100	AAA79321
ORF17	23751.24347	ORF1	ORF1	*Aeromonas hydrophila*	100	AAA79320
ORF18	24344.25090	ORFJ, partial	ORFJ	*Aeromonas hydrophila*	99	AAA79319
ORF19	25284.26306	general secretion pathway protein K	*gspK*	*Pseudomonas stutzeri* DSM 4166	49	YP_002798801
ORF20	26306.26911	type II secretion system protein	*gspJ2*	*Azotobacter vinelandii* DJ	48	YP_002798802
ORF21	26908.27312	type II secretion system protein	*gspJ1*	*Pseudomonas stutzeri* DSM 4166	58	AEA85764
ORF22	27306.27677	type II secretion system protein	*gspG2*	*Pseudomonas stutzeri* DSM 4166	48	AEA85763
ORF23	27680.28114	General secretion pathway protein G	*gspG1*	*Pseudomonas stutzeri* DSM 4166	84	AEA85762
ORF24	28133.29332	type II secretion system protein	*gspF*	*Azotobacter vinelandii* DJ	60	YP_002798806
ORF25	29332.30987	type II secretion system protein E	*gspE*	*Azotobacter vinelandii* DJ	76	YP_002798807
ORF26	31755.32303	dTDP-4-dehydrorhamnose 3,5-epimerase	*rmlC*	*Escherichia* *sp.* TW09308	81	ZP_09461002
ORF27	32368.33246	glucose-1-phosphate thymidylyltransferase	*rmlA*	*Aeromonas salmonicida* subsp. *salmonicida* A449	94	YP_001141271
ORF28	33359.34246	dTDP-4-dehydrorhamnose reductase	*rmlD*	*Aeromonas hydrophila* subsp. *hydrophila* ATCC 7966	98	YP_857411
ORF29	34246.35331	dTDP-glucose 4,6-dehydratase	*rmlB*	*Aeromonas hydrophila* subsp. *hydrophila* ATCC 7966	97	YP_857412
ORFd	36022.37437	outer membrane protein OprM	*oprM*	*Aeromonas hydrophila* subsp. *hydrophila* ATCC 7966	98	YP_857413

The O-antigen gene cluster of AL06-01 and AL06-06 contain six and four different glycosyltransferase genes, respectively, that are required for the assembly of nucleotide sugar repeat on the membrane lipid undecaprenol pyrophosphate (Und-PP) (Tables 4 and 5). AL06-06 O-antigen clusters, unlike the VAh O-antigen cluster, was predicted to contain the *wbtB* gene that encodes undecaprenyl-phosphate alpha-N-acetylgalactosaminyl 1-phosphate transferase required for the transfer of the GalNAc-1-phosphate moiety from UDP-GalNAc onto the carrier lipid undecaprenyl phosphate (Figure 5). In contrast, the O-antigen cluster of AL06-01, like the VAh O-antigen cluster, contains the *wecA* gene that encodes undecaprenyl-phosphate alpha-N-acetylglucosaminyl 1-phosphate transferase required for the transfer of the GlcNAc-1-phosphate moiety from UDP-GlcNAc onto the carrier lipid undecaprenyl phosphate (Figure 5).

A genome-wide comparison of the RAh isolates AL97-91 and MN98-04 using BLAST matrix showed that they are highly similar (>94%) in terms of their conserved gene families (Figure 3). Both the isolates were predicted to contain an O-antigen biosynthesis gene cluster that was highly similar to each other in terms of gene content, relative organization of the genes and the percent identity of their predicted gene products (Table 6 and Figure 5). The O-antigen biosynthesis gene clusters of RAh isolates AL97-91 and MN98-04 contain 15 ORFs predicted to be involved in O-antigen biosynthesis, including gene products predicted to be required for the biosynthesis of dTDP-rhamnose and D-glucose (Table 6). They contain six different glycosyltransferase genes and one acetyl transferase gene in their O-antigen clusters (Table 6). Additionally, they contain the *wecA* gene required for the transfer of the GlcNAc-1-phosphate moiety from UDP-GlcNAc onto the carrier lipid undecaprenyl phosphate. The absence of an O-antigen polymerase gene within those clusters suggests these two isolates may produce a semi-rough O-antigen. The analysis of the O-antigen biosynthesis gene cluster of strains AL97-91 and MN98-04 demonstrated that these two strains contain two additional cluster of genes required for S-layer protein synthesis and type II secretion in their O-antigen clusters (Table 6 and Figure 5). These results suggest the O-antigen of these two strains anchor the S-layers and most probably the S-layers of *A. hydrophila* isolates AL97-91 and MN98-04 are secreted by a type II secretion system, unlike in *Caulobacter crescentus* that secretes S-layer proteins via a type I secretion system [45]. 

All together we have identified four unique O-antigen biosynthesis clusters among the 11 sequenced *A. hydrophila* strains (Figure 5) and this increases the number of known types of O-antigen biosynthesis clusters in *A. hydrophila* to a total of 7 (Figure 4). The diversity of O-antigen biosynthesis clusters in *A. hydrophila* isolates also suggests the possible contribution of LGT events. The nucleotide sequences of O-antigen biosynthesis gene clusters from 11 *A. hydrophila* strains sequenced in this study are deposited in GenBank with accession nos. KC999966 and KC999968 to KC999977.

### Epidemic-associated genomic islands (GIs)

**Figure 6:**
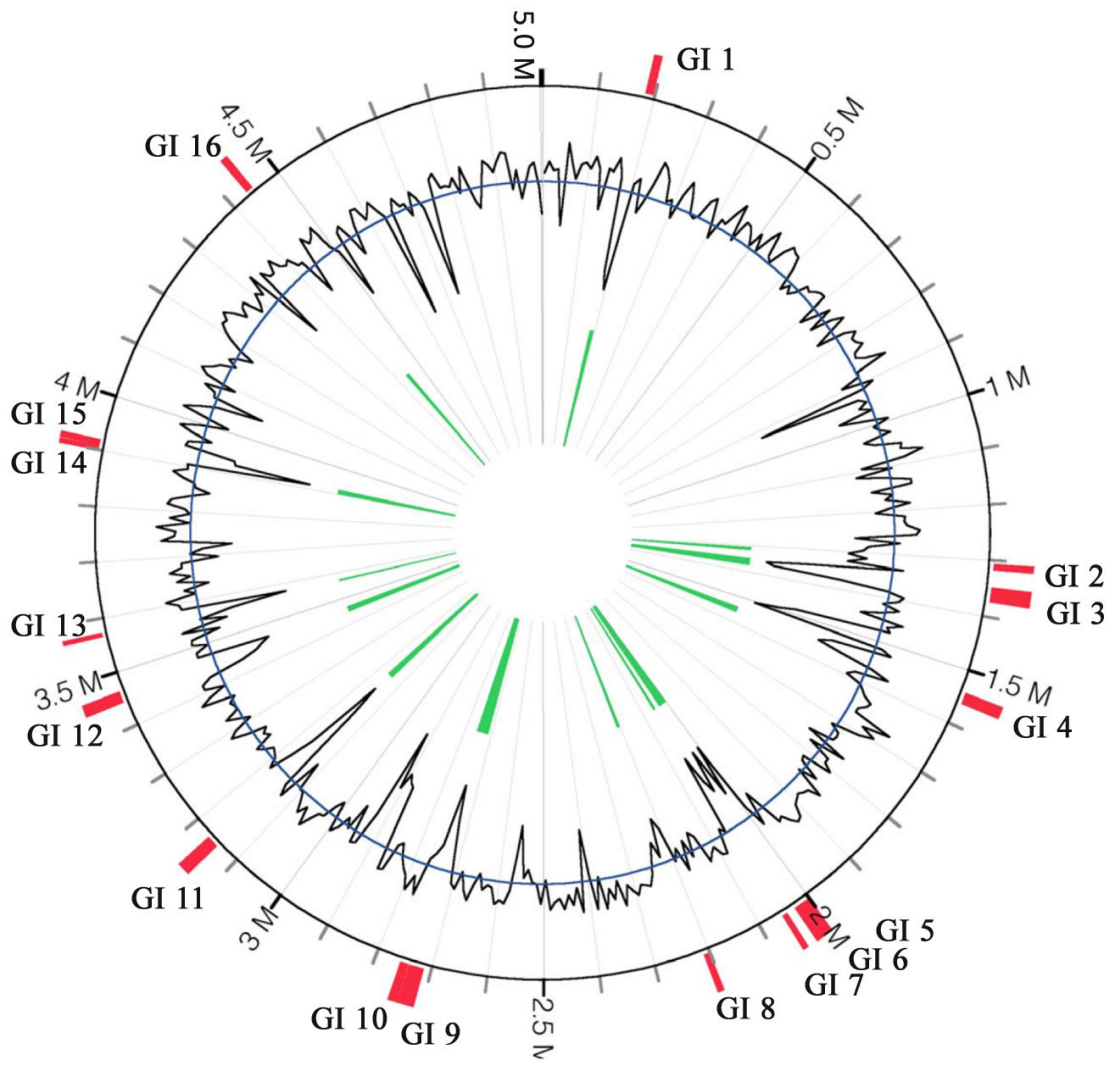
Predicted genomic islands (GIs) within the genome of *A. hydrophila* ML09-119. GIs were predicted using the IslandViewer tool [32]. The black line indicates the %G+C content. All of the predicted GIs showed a %G+C content bias much lower than the average %G+C content of *A. hydrophila* (61.0%).

Since genomic islands contribute to lateral gene transfer and bacterial evolution [46], we analyzed the epidemic *A. hydrophila* isolates for the presence of genomic islands. We identified 16 GIs, ranging from 8 kb to 30 kb and comprised of 252.45 kb that encode 255 ORFs (Dataset S8), within the genomes of VAh isolates (Figure 6). Nine of the GIs were considered as epidemic-associated GIs since they were absence from the RAh isolates (Table 7). The nucleotide sequences for each of the GIs found within the genome of VAh type strain ML09-119 are provided in Dataset S7. The GI 2 region contains a cluster of genes involved in *myo*-inositol catabolism. GI 3, largest among the nine epidemic-associated unique GIs with 25 ORFs, includes genes coding hypothetical proteins, proteins involved in thiamine and cobyric acid biosynthesis and RNA metabolism. GI 12 includes a type I restriction modification system, DNA helicase, DNA repair protein, anticodon nuclease, as well as transposases (T7 like) and regulatory proteins along with hypothetical proteins of unknown function (Dataset S8). This GI is predicted to be generated in VAh isolates after T7 transposition since a GI with these fitness-enhancing features is generated after integrating at an *attTn7* site within a bacterial genome [47]. GI 13 encodes a CS5 pilus biogenesis cluster (Table 8) which is similar to that of the enterotoxigenic *E. coli* O115:H40 [48]. GI 16 of VAh isolates is also predicted to contain a cluster of genes required for pilus biogenesis.

**Table 7:** The distribution of 16 different VAh-specific genomic islands in different *A. hydrophila* isolates used in this study.

GI #	Nucleotide positions	Size (kb)	Number of ORFs	%G+C	*A. hydrophila* isolates	Epidemic-associated GIs
GI 1	8728.22581	13,853	12	44.0	ML09-119, ML09-122, ML09-121, AL09-79, AL10-121, PB10-118	Unique
GI 2	30195.41895	11,700	13	57.8	ML09-119, ML09-122, ML09-121, AL09-79, AL10-121, PB10-118	Unique
GI 3	71487.98770	27,283	28	41.3	ML09-119, ML09-122, ML09-121, AL09-79, AL10-121, PB10-118	Unique
GI 4	263720.284778	21,058	32	54.0	ML09-119, ML09-122, ML09-121, AL09-79, AL10-121, PB10-118, AL97-91, MN98-04, TN97-08, AL06-01	-
GI 5	76843.95300	18,457	22	51.5	ML09-119, ML09-122, ML09-121, AL09-79, AL10-121, PB10-118, AL97-91, MN98-04, TN97-08, AL06-01	-
GI 6	95365.107861	12,496	12	48.1	ML09-119, ML09-122, ML09-121, AL09-79, AL10-121, PB10-118	Unique
GI 7	123898.133969	10,071	12	44.5	ML09-119, ML09-122, ML09-121, AL09-79, AL10-121, PB10-118, TN97-08, AL06-01	-
GI 8	277559.288667	11,108	13	54.2	ML09-119, ML09-122, ML09-121, AL09-79, AL10-121, PB10-118, AL97-91, MN98-04	-
GI 9	91274.118831	27,557	36	51.1	ML09-119, ML09-122, ML09-121, AL09-79, AL10-121, PB10-118, TN97-08	-
GI 10	119893.134877	14,984	20	57.4	ML09-119, ML09-122, ML09-121, AL09-79, AL10-121, PB10-118, TN97-08	-
GI 11	21645.43624	21,979	33	42.3	ML09-119, ML09-122, ML09-121, AL09-79, AL10-121, PB10-118	Unique
GI 12	25686.46472	20,787	19	49.9	ML09-119, ML09-122, ML09-121, AL09-79, AL10-121, PB10-118	Unique
GI 13	1.8099	8,098	12	35.2	ML09-119, ML09-122, ML09-121, AL09-79, AL10-121, PB10-118	Unique
GI 14	51060.60399	9,339	12	63.6	ML09-119, ML09-122, ML09-121, AL09-79, AL10-121, PB10-118	Unique
GI 15	60579.71065	10,486	13	43.6	ML09-119, ML09-122, ML09-121, AL09-79, AL10-121, PB10-118, TN97-08,	-
GI 16	351791.364972	13,182	18	56.4	ML09-119, ML09-122, ML09-121, AL09-79, AL10-121, PB10-118	Unique

**Table 8:** Summary of ORFs encoded within GI 13 of EAh isolate ML09-119 involved in CS5 pilus biogenesis.

ORF ID	Nucleotide positions in contig 27	Putative function	Gene	Top BLASTx hit	% Identity	E-value	% Identity to *E. coli* (Accession no) [48]
ORF1	106.456	Extracellular solute-binding protein family 3	-	*Paenibacillus lactis* 154	48	2.27917	-
ORF2	909.1490	CS5 fimbrial major pilin subunit	*hsfA*	*Escherichia coli*	38	2E-24	38 (CAA11820)
ORF3	1555..2247	25.9 kDa protein in CS5 3' region precursor	*hsfB*	*Edwardsiella ictaluri* 93-146	36	3.82E-30	28 (CAA11821)
ORF4	2303.3643	P pilus assembly protein porin PapC-like protein	*hsfC*	*Edwardsiella ictaluri* 93-146	29	9.65E-45	25 (CAA11822)
ORF5	3945.4679	P pilus assembly protein porin PapC-like protein	*hsfC*	*Edwardsiella ictaluri* 93-146	28	6.78E-19	22 (CAA11822)
ORF6	4669.5244	Hypothetical protein	*hsfE*	*Escherichia coli*	26	4.91E-08	26 (CAA11823)
ORF7	5205.6023	CS5 fimbrial minor pilin subunit	*hsfD*	*Escherichia coli*	35	2.56E-19	35 (CAA11825)
ORF8	6369.7127	EAL domain protein	-	*Vibrio parahaemolyticus* AN-5034	45	1.92E-56	-
ORF9	7258.8034	Alpha/beta hydrolase, putative	-	*Vibrio cholerae* MZO-3	49	3.35E-64	-

### Virulence factors in epidemic-associated unique regions

**Figure 7:**
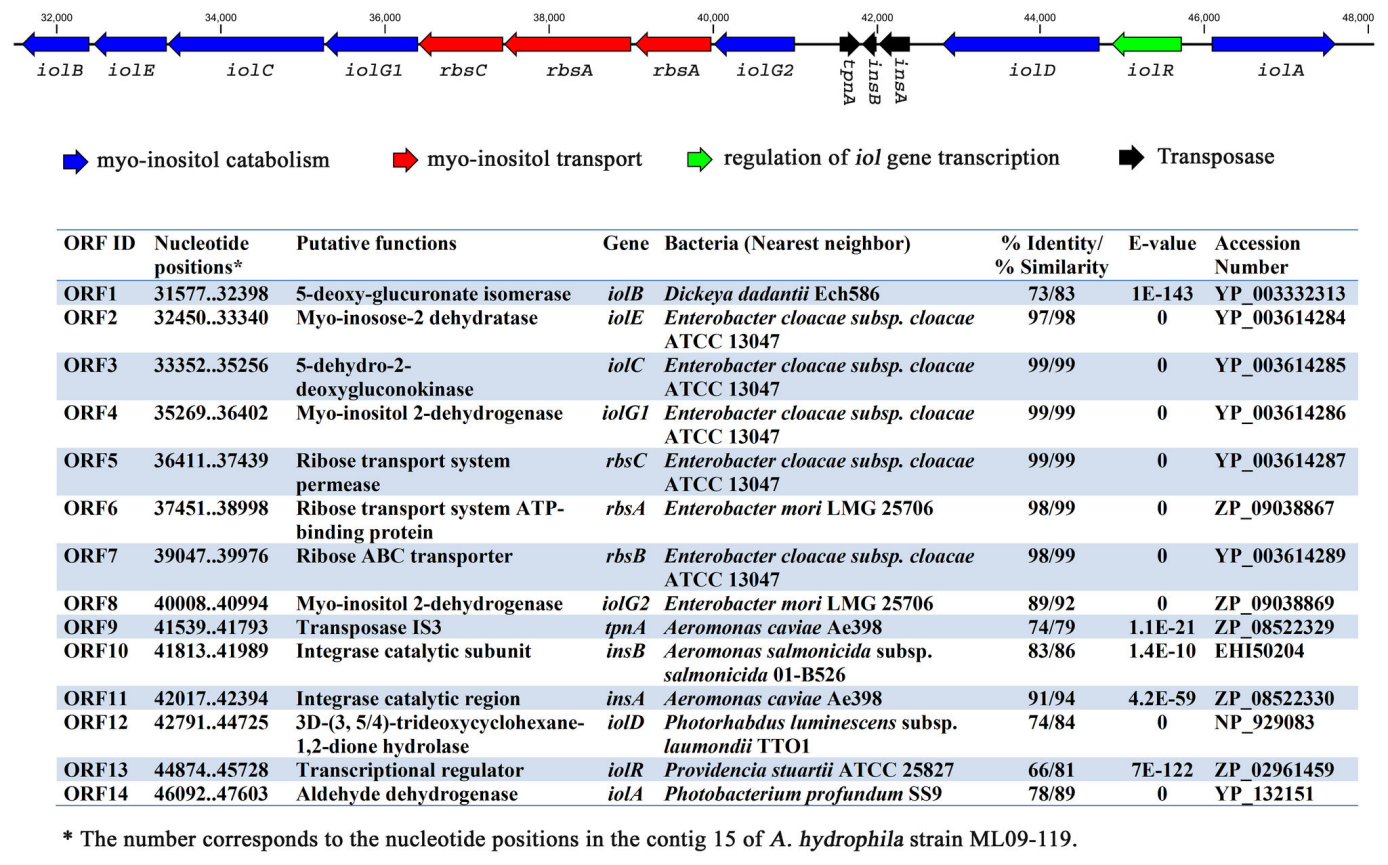
Genetic elements involved in *myo-*inositol utilization in VAh strains. The schematic organization depicts the cluster of genes involved in *myo*-inositol utilization in epidemic *A. hydrophila* ML09-119. The presence of a functional *myo*-inositol utilization pathway in VAh strains was confirmed by their ability to grow on *myo*-inositol as a sole carbon source.

The highly virulent nature of the recent epidemic *A. hydrophila* isolates [4] and the presence of most of the predicted virulence factors within genomic islands [49] prompted us to search for virulence factors within the epidemic-associated genomic regions of epidemic *A. hydrophila*. We found 34 predicted virulence factors within the epidemic-associated unique regions of VAh isolates (Table 9). The average percent identity of those proteins to their homologous virulence factors was 38 %. We found that 35% (12 out of 34) of the virulence factors were located within the GIs, which is in agreement with the common occurrence of virulence factors within genomic islands [49]. Genes predicted to encode a fimbrial major subunit and fimbrial usher, and fimbrial chaperon were found within GI 13 and GI 16, respectively. Two putative TonB-dependent receptor coding genes were identified within the epidemic-associated unique regions of VAh isolates, with one showing 41% homology to a TonB-dependent receptor in *Neisseria meningitidis* MC58 (serogroup B) and the other with 26% homology to a Yersinia bactin receptor protein of *Yersinia pestis* CO92 (Table 9). Three genes (*iolG*, *rbsA* and *iolA*) located within the *myo*-inositol utilization cluster (Figure 7), which is also part of GI 2, were predicted to encode virulence-related proteins (Table 9). In GI 12, we found *hsdR* and *hsdM* of a putative type I restriction modification system that share 25% and 24% identity to their homologs in *Vibrio cholerae* N1696, respectively (Table 9). Guanylate cyclases, involved in bacterial cell division, motility, biofilm formation and pathogenesis [50], were predicted within the epidemic-associated unique regions C32R2 and C27R1. Putative virulence factors found within the epidemic-associated regions could potentially contribute to enhance pathogenicity of VAh strains. 

**Table 9:** Predicted genes that have homology to putative virulence factors and are present within VAh-associated genomic regions.

Unique region ID	VFDB ID	GIs	Gene	Putative functions	Organisms	% Identity	E-value
C8R1	VFG1693	GI 1	*int*	Prophage P4 integrase	*Escherichia coli* CFT073	33	1.1E-45
C10R1	VFG0893	-	*papA_2*	PapA protein	*Escherichia coli* CFT073	33	2.1E-16
C10R1	VFG0075	-	*InlA*	Internalin A	*Listeria monocytogenes* (serovar 1/2a) EGD-e	30	1.4E-16
C13R2	VFG0783	-	*intL*	Putative integrase for prophage 933L and the LEE pathogenicity island	*Escherichia coli* O157:H7 EDL933	27	1.3E-16
C15R4	VFG0038	GI 2	*bplA*	probable oxidoreductase	*Bordetella pertussis* Tohama I	28	2.3E-19
C15R4	VFG0344	GI 2	*hitC*	iron(III) ABC transporter, ATP	*Haemophilus influenzae* Rd	33	6.7E-23
C15R5	VFG0082	-	*aldA*	aldehyde dehydrogenase	*Vibrio cholerae* N16961	31	1.2E-47
C15R6	VFG0598	-	*intC*	Sai integrase	*Shigella flexneri* (serotype 2a) 301	47	4.9E-93
C18R3	VFG0672	GI 6	*int*	integrase	*Shigella flexneri* (serotype 2a)	66	5E-162
C20R4	VFG1124	-	*VC1791*	conserved hypothetical protein	*Vibrio cholerae* N16961	41	4.2E-39
C20R7	VFG0925	-	*fepC*	Ferric enterobactin transport ATP	*Escherichia coli* CFT073	33	2.3E-23
C20R7	VFG0922	-	*chuU*	Putative permease of iron compound ABC transport system	*Escherichia coli* CFT073	39	3.8E-29
C20R8	VFG0358	-	*fyuA/psn*	yersiniabactin receptor protein	*Yersinia pestis* CO92	26	1.4E-34
C20R8	VFG0167	-	*pchR*	transcriptional regulator PchR	*Pseudomonas aeruginosa* PAO1	41	1.1E-26
C26R1	VFG1102	GI 12	*hsdM*	DNA methylase HsdM	*Vibrio cholerae* N16961	25	4.6E-17
C26R1	VFG1098	GI 12	*hsdR*	type I restriction enzyme HsdR	*Vibrio cholerae* N16961	24	1.3E-21
C26R2	VFG2417	-	*ecpE*	hypothetical protein	*Escherichia coli* O157:H7 EDL933	47	5.7E-41
C26R2	VFG2416	-	*ecpD*	putative receptor	*Escherichia coli* O157:H7 EDL933	56	1.2E-108
C26R2	VFG2415	-	*ecpC*	putative enzyme	*Escherichia coli* O157:H7 EDL933	55	8.5E-142
C26R2	VFG2412	-	*ecpB*	hypothetical protein	*Escherichia coli* O157:H7 EDL933	48	1.1E-50
C26R2	VFG2414	-	*ecpA*	hypothetical protein	*Escherichia coli* O157:H7 EDL933	57	7.2E-44
C26R2	VFG2044	-	*bvgA*	Virulence factors transcription regulator	*Bordetella pertussis* Tohama I	47	7.7E-53
C27R1	VFG1433	GI 13	*csvA*	CS7 fimbria major subunit CsvA precursor	*Escherichia coli*	39	6.8E-22
C27R1	VFG0584	GI 13	*yjcC*	putative diguanylate cyclase	*Salmonella enterica* (serovar *typhimurium*) LT2	29	4.8E-28
C32R1	VFG1584	GI 14	*orf50*	hypothetical protein	*Escherichia coli* 536	26	1.9E-13
C32R1	VFG1584	GI 14	*orf50*	hypothetical protein	*Escherichia coli* 536	26	4.7E-18
C32R2	VFG1888	-	*letS*	sensory box histidine kinase	*Legionella pneumophila* Philadelphia 1	39	1.1E-41
C32R2	VFG0584	-	*yjcC*	putative diguanylate cyclase	*Salmonella enterica* (serovar typhimurium) LT2	32	6.9E-32
C36R3	VFG1092	GI 16	*int3*	integrase, phage family	*Vibrio cholerae* N16961	26	9.3E-15
C36R3	VFG1443	GI 16	*ompA*	outer membrane protein A	*Escherichia coli*	38	1.8E-54
C36R3	VFG1548	GI 16	*prfC*	PrfC protein	*Escherichia coli* 536	39	1E-164
C36R3	VFG1547	GI 16	*prfD*	PrfD protein	*Escherichia coli* 536	51	2.7E-61
C39R1	VFG0266	-	*hmbR*	hemoglobin receptor	*Neisseria meningitidis* MC58 (serogroup B)	31	1.4E-45
C39R1	VFG0266	-	*hmbR*	hemoglobin receptor	*Neisseria meningitidis* MC58 (serogroup B)	36	6.5E-34

### VAh strains contain a *myo*-inositol utilization pathway

The comparative analysis of *A. hydrophila* genomes revealed that a 17.5 kb genomic region predicted to be involved in *myo*-inositol catabolism is present in all VAh isolates and is part of the epidemic-associated region in VAh isolates (Dataset S6). Consistent with this finding, it was observed that all VAh isolates were able to use *myo*-inositol as a sole carbon source. Neither the five RAh strains nor the ATCC 7966 reference proteome [19] was predicted to contain genetic regions involved in *myo*-inositol catabolism. These findings were supported by the inability of any RAh isolate to use *myo*-inositol as a sole carbon source. The 17.5 kb *myo*-inositol catabolism cluster (*iol*) contains 11 ORFs that are predicted to be involved in *myo*-inositol transport and catabolism (Figure 7). In VAh strains, like *Bacillus subtilis* [51], *myo*-inositol catabolism and transport genes are clustered together within a single region whereas in some bacteria, including *Corynebacterium glutamicum* [52] and *Caulobacter crescentus* [53], these genes are split into two or more clusters and dispersed across the chromosome. The comparison of the *iol* cluster of VAh isolates with that of *Bacillus subtilis* [54,55] and *Klebsiella* (Aerobacter) *aerogenes* [56] revealed that the VAh *myo*-inositol catabolism pathway cluster encodes all of the enzymes necessary for *myo*-inositol utilization with the exception of 2-deoxy-5-keto-D-gluconic acid 6-phosphate aldolase, which is required for the degradation of *myo*-inositol to acetyl-CoA (Figure 7). However, a search in the ML09-119 genome did reveal a gene predicted to encode a homolog of 2-deoxy-5-keto-D-gluconic acid 6-phosphate aldolase, with 98% similarity to its nearest BLASTx hit within the genome of *A. hydrophila* ATCC 7966. 

The low %G+C content of the region encoding the *iol* cluster was 56.2% compared to the average 60.9 %G+C content of the entire genome and the presence of a transposase flanking the *iol* cluster suggest that the genetic region encoding the *myo*-inositol catabolism genes has been introduced into the VAh genome via a LGT event(s) (Figure 7). It is noteworthy to mention that ORF1 to ORF11 of the *myo*-inositol catabolism gene cluster is part of GI 2 present in an epidemic-associated region (Dataset S6).

### Establishment of genotypic and phenotypic tests to identify epidemic *A. hydrophila* strains

The clonal nature of sequenced VAh isolates prompted us to develop a VAh-specific PCR, and to compare results from the PCR-based detection of VAh strains with the ability of each strain to use *myo*-inositol as a sole carbon source. A multiplex PCR was carried out with the *gyrB* gene as an internal positive control and the VAh-associated C13R2-specific primers to screen 68 *A. hydrophila* isolates obtained from diseased catfish as well as other presumably RAh isolates from pond sediment or fish cloaca with no evidence of MAS. RAh isolates and *A. hydrophila* ATCC 7966, which also served as a negative control, did not produce any amplicon specific to C13R2 region while the *gyrB* gene specific PCR was positive. Among 68 *A. hydrophila* isolates tested, 47 isolates (69%) were positive for the C13R2-specific PCR and all were positive for the *gyrB* gene specific PCR. 

The presence of genetic loci involved in *myo*-inositol catabolism in all sequenced VAh isolates prompted us to determine the ability of *A. hydrophila* isolates to use *myo*-inositol as a sole carbon source. We tested the same 68 *A. hydrophila* isolates (evaluated above for C13R2 region-specific PCR) for their growth in M9 minimal medium containing *myo*-inositol. Of the 68 isolates, the same 47 isolates that were positive by C13R2-specific PCR were also capable of using *myo*-inositol as a sole carbon source. Taken together, these results demonstrated that 100% of the isolates showed a correlation between the growth on *myo*-inositol and the presence of an epidemic-associated region. 

### Predicted prophages within the genome of *A. hydrophila* isolates

Prophages contribute significantly to the evolution of their bacterial hosts [57]. We predicted 5 prophages (AH1, AH2, AH3, AH4 and AH5) within the genome of VAh isolates and the distribution of these prophages in *A. hydrophila* genomes sequenced in this study are presented in Table 10. Among the five prophages predicted in all VAh isolates, prophage AH1 and AH5 were uniquely present in all VAh isolates and absent from all other RAh isolates (Table 10). 

**Table 10:** Distribution of five prophages in different *A. hydrophila* isolates used in this study.

*A. hydrophila* isolates	AH1	AH2	AH3	AH4	AH5
AL06-06	Absent	Partial	Present	Absent	Absent
AL06-01	Absent	Absent	Absent	Absent	Absent
AL97-91	Absent	Partial	Partial	Partial	Absent
TN97-08	Absent	Partial	Partial	Absent	Absent**^*a*^**
MN98-04	Absent	Present	Partial	Partial	Absent
ML09-119	Present	Present	Present	Present	Present
ML09-121	Present	Present	Present	Present	Present
ML09-122	Present	Present	Present	Present	Present
AL09-79	Present	Present	Present	Present	Present
AL10-121	Present	Present	Present	Present	Present
PB10-118	Present	Present	Present	Present	Present

*a* The reference mapping of TN97-08 against the prophage AH5 found that some reads from TN97-08 matched with this prophage genome but none of them encoded complete ORFs.

The putative prophage AH1, found only in VAh isolates, showed the highest number of protein similarities to Fels 1 prophage of *Salmonella*. This prophage is 17.5 kb in size and encodes a total of 16 different predicted ORFs. This prophage is predicted as a questionable prophage due to the deficiency of some structural proteins. Prophage AH1 encodes two different transferrin-binding proteins and these proteins might help epidemic VAh strains to acquire iron, an essential cofactor for diverse biochemical reactions, from carrier protein transferrin. Two transferrin-binding proteins located within the AH1 prophage correspond to ORF7 and ORF8 of the epidemic-associate unique region C39R1 (Dataset S6). Prophage AH1 also is predicted to encode a methyl-accepting chemotaxis protein. In *Pseudomonas aeruginosa*, it has been shown that the methyl-accepting chemotaxis protein plays a significant role in the regulation of virulence and antibiotic tolerance [58].

The putative prophage AH2, present in all VAh strains and all RAh except AL06-01, shares significant homology with phiO18P of *A. media* [59] and phiO18P-like prophages of *A. caviae* Ae398 [60] and *A. salmonicida* subsp. *salmonicida* A449 [61]. AH2 was found to be integrated into the tRNA-Leu gene at an *attL* site on the VAh genome. The sequence analysis demonstrated that this 35 kb putative prophage is flanked by 50-bp direct repeats, predicted as attachment sites *attL* and *attR*. The analysis of CDSs from prophage AH2 indicated that most of them have significant homology with that of prophage phiO18P, a UV-induced phage from *A. media* isolate O18 [59]. The *attL* site of prophage AH2 is located within the tRNA-Leu gene like many other prophages inserted within bacterial genomes [62]. Similarly, the disruption of a tRNA-Leu gene with a phiO18P-like prophage was also observed in *A. caviae* Ae398. The %G+C content of the AH2 prophage is 58.0%. A prophage database compiled by Zhou et al. [61] demonstrated that *A. salmonicida* subsp. *salmonicida* A449 contained two putative prophages similar to phiO18P with a 58% G+C content. A comparative genome sequence analysis from prophage AH2, phiO18P [59], and two prophages from *A. salmonicida* subsp. *salmonicida* A449 demonstrated a high degree of sequence homology among these prophages (data not shown). 

The putative prophage AH3 has many proteins that are homologous with those from prophage *Salmonella* RE-2010 and is located within the genome of all VAh strains. This prophage was absent in all other RAh strains except AL06-06. The AH3-like prophage of RAh strain AL06-06 is 35.7 kb in size and encodes a total of 58 ORFs whereas the prophage AH3 of VAh strains is 7.8 kb in length with 12 predicted ORFs. The 47.93% G+C content of this prophage is much lower than the %G+C content of *Aeromonas* species. The smaller size of this incomplete prophage and its lower %G+C content as compared to that of the RAh strain AL06-06 suggests that this prophage might have undergone several LGT events.

**Figure 8:**
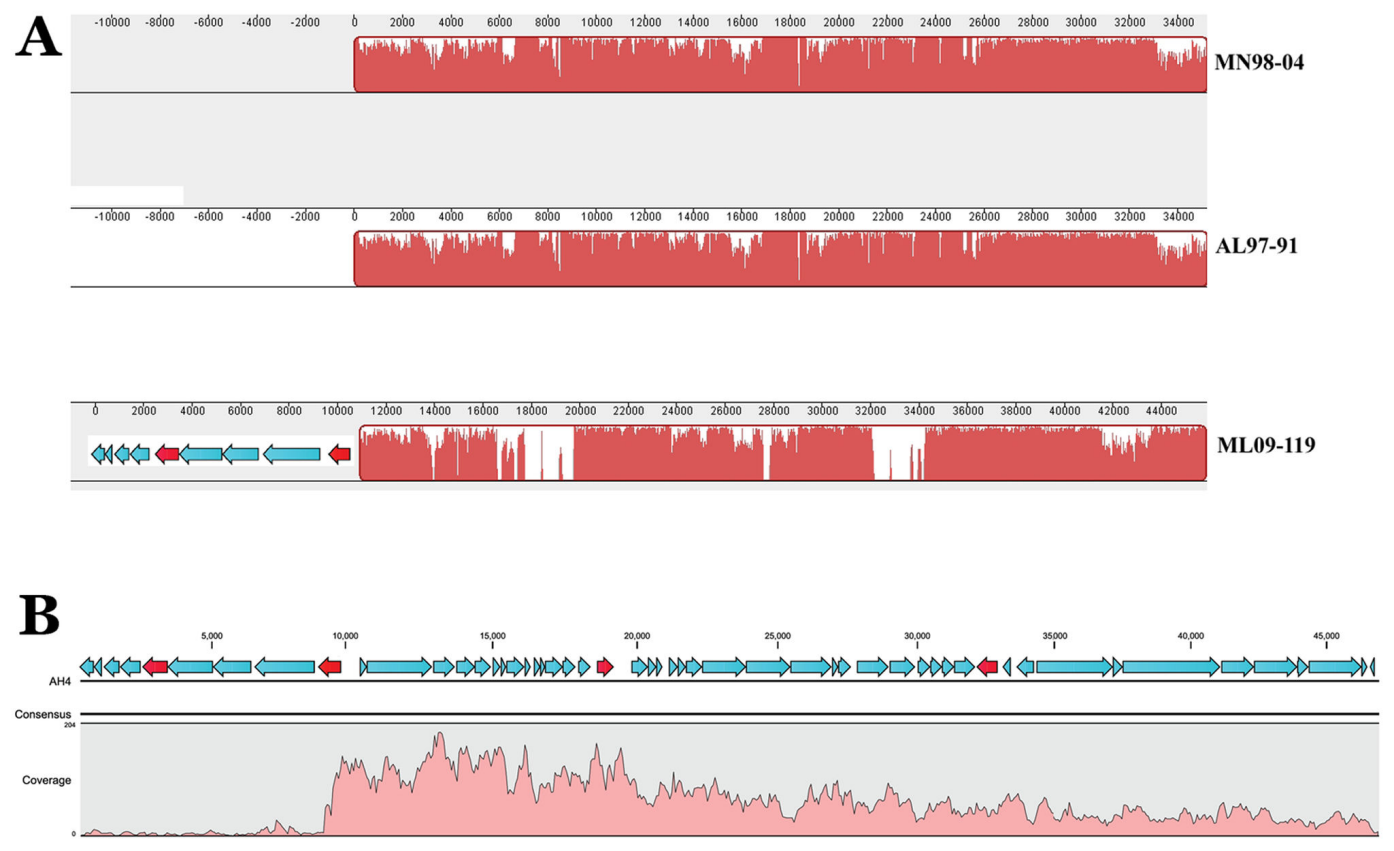
Identification of a VAh-specific genetic region associated with prophage AH4 that is induced after mitomycin C treatment. (Panel A) Mauve multiple genome alignment of the prophage A4 region from AH strains MN98-04 and AL97-91 with VAh strain ML09-119, depicting the two upstream ORFs (in red) and two within-prophage regions (shown by lack of Mauve alignment) that are VAh-associated. (Panel B) Induced phage DNAs were subjected to 454 pyrosequencing and were reference mapped against the AH4 prophage region of the *A. hydrophila* Ml09-119 genome. Each predicted ORF is indicated as an arrow, and the four VAH-associated ORFs are depicted in red.

Prophage AH4 (46.9 kb in size with 53 ORFs) was observed within the genome of all VAh isolates and showed significant homology to the Mu-like prophage D108 of *Escherichia coli* origin [63]. The 57.3% G+C content of this prophage is similar to that of *Aeromonas salmonicida* subsp. *salmonicida* A449 [64]. None of the ORFs from this prophage has any known affiliation with any previously described *A. hydrophila* phage. The M and S subunits of a type I restriction modification system and secretion activator proteins encoded in this AH4 prophage could be potential virulence factors in *A. hydrophila*. The analysis of predicted prophages within the 6 RAh *A. hydrophila* isolates sequenced in this study demonstrated that isolates AL97-91 and MN98-04 contain AH4-like prophages with significant homology to Mu-like prophage D108 of Escherichia coli origin [63]. The sizes of the AH4-like prophages from AL97-91 and MN98-04 isolates are 37.5 kb, unlike AH4 prophages of VAh isolates which is 46.9 kb. The comparison of the AH4 prophage from VAh strains with the AH4-like prophages of MN98-04 and AL97-91 demonstrated that AH4 from VAh strains contains at least 10.6 kb of additional sequences that are absent in AH4-like prophages of MN98-04 and AL97-91 (Figure 8A). An additional sequence present in the AH4 prophage encodes 10 different ORFs and two of them are present in epidemic-associated regions (ORFs 1 from C18R3 and C18R4, Dataset S6). This prophage also contains two additional epidemic-associated regions (C18R5 and C18R6, Dataset S6) that are clearly evident from the mauve alignment of this prophage region from VAh isolates and the AH4-like prophage from the RAh strains MN98-04 and AL97-91 isolates (Figure 8A).

Prophage AH5, found within the VAh isolates but absent from RAh isolates, shows the highest number of protein similarities to Enterobacterial phage mEp390. This epidemic-associated prophage is 33.1 kb in length and encodes 40 predicted ORFs. The %G+C content of this prophage is 52.19% which is much lower than the average %G+C content (61%) of *A. hydrophila* isolates. AH5 is predicted to encode a N^6^-methyltransferase and this could potentially contribute to *A. hydrophila* virulence in catfish due to the virulence properties of this protein in many bacteria including *A. hydrophila* [65,66].

### Identification of induced prophage by 454 pyrosequencing

Phage DNA was recovered from the cell-free supernatant of a mitomycin C-induced ML09-119 culture, and the phage DNA was isolated to determine which of the prophage(s), out of the five predicted prophages, were induced after mitomycin C treatment. Purified prophage DNAs visualized by pulsed field agarose gel electrophoresis revealed only a single DNA band (data not shown). This observation suggested that only one prophage was induced from *A. hydrophila* ML09-119 after mitomycin C treatment. Induction of VAh isolate ML09-119 followed by sequencing of phage DNA and reference mapping of the phage sequences against the ML09-119 genome suggests that AH4 is the only inducible prophage in VAh isolates (Figure 8B). This prophage was predicted as prophage AH4 with significant similarity to *Escherichia*_phage_D108 (Table 10). Induced phage particles were visualized with electron microscopy revealing a phage morphology with an icosahedral head and a contractile tail. These results strongly suggest that *A. hydrophila* ML09-119, and presumably all other VAh strains, contain a lysogenic phage. The sequence coverage that mapped to prophage AH4 was greater than 50× on average (Figure 8B). This reference assembly data indicated the presence of a single inducible prophage, which was in agreement with the previous observation of a single DNA band observed by pulsed field gel electrophoresis. In addition, there were 4 ORFs (two upstream and two within the prophage) associated with the AH4 prophage that were only present in sequenced VAh strains, and the upstream ORFs were not induced by mitomycin C as strongly as the prophage region. The two upstream epidemic-associated ORFs were predicted to encode an Abi family protein and a Cro/CI family transcriptional regulator (Figure 8A), with the putative Cro/CI transcriptional regulator located very close to the induced region of the prophage. The two epidemic-associated ORFs within the induced prophage were both predicted to encode hypothetical proteins of unknown function. Given the well described contribution of transduced loci to bacterial pathogenesis in *V. cholera* and other pathogens, it is conceivable that these AH4 prophage-associated genes are involved in lysogenic conversion of VAh strains into a more virulent phenotype. Further studies will be necessary to determine the specific contribution of these genetic loci, and other VAh-associated loci, to the pathogenesis of these *A. hydrophila* strains responsible for epidemic outbreaks in farmed catfish.

## Discussion

This study identified specific genetic loci present within *A. hydrophila* isolates responsible for an epidemic of disease in catfish. Comparative genomics of 11 *A. hydrophila* isolates demonstrated that recent epidemic isolates are highly clonal while a great deal of diversity was observed among *A. hydrophila* isolates obtained from diseased fish prior to any epidemic outbreak. Recent VAh isolates have considerable genomic differences with RAh strains that may contribute to their emergence as highly pathogenic strains in aquaculture farmed catfish.

The recent epidemic outbreak of MAS caused by highly virulent *A. hydrophila* is unique since the catfish farming operations in the Southeastern United States have never experienced a large-scale outbreak of MAS previously [3]. Moreover, experimental disease challenges in aquaria models indicate that the *A. hydrophila* isolates responsible for recent epidemic outbreaks are highly virulent as compared to the RAh isolates that were historically regarded as an opportunistic bacterial pathogen isolated from stressed fish [4]. Since after introduction into catfish farming in western Alabama in 2009, this unprecedented epidemic has expanded its geographic territory and caused frequent outbreaks in the summer months, resulting in millions of pounds of losses in Alabama, Mississippi and Arkansas.

Epidemic isolates used in our study were obtained as pure cultures from tissues (kidney or brain) taken from diseased catfish in ponds experiencing an outbreak of MAS. Our comparative genomics data distinguishes these contemporary epidemic isolates (VAh strains) from reference isolates (RAh strains) based on the presence of specific genetic polymorphisms. Our findings suggest that those epidemic-associated genetic markers have been acquired during the course of evolution of highly virulent strains. Our comparative genomic analysis demonstrated that all of the VAh isolates contain a large number of unique regions within their genomes that are completely absent in the genomes of RAh isolates. A total of 55 regions comprising 336,469 bp were identified as epidemic-associated regions in VAh isolates. These VAh-associated genomic regions all together encode 313 ORFs that are predicted to be involved in different functions. A large fraction of these VAh-associated genomic regions (252 kb out of 336 kb in total) are contained within genomic islands, suggesting their possible acquisition through lateral transfer. A total of 34 predicted genes that had significant similarity with proteins in a virulence factor database were predicted within these VAh-associated regions. Further experiments will be required to determine the specific contribution of each genetic locus to VAh virulence in catfish.

A cluster of genes (*iol*) involved in *myo*-inositol catabolism and transport were consistently present in all sequenced VAh isolates, and were not identified in any other *A. hydrophila* genome. This is the first identification of an *iol* cluster, which encodes all of the proteins required for the transport and catabolic degradation of *myo*-inositol to acetyl-CoA, within any *Aeromonas* species. The existence of a functional *myo*-inositol catabolism pathway in epidemic-associated *A. hydrophila* isolates was formally demonstrated by the ability of these strains to grow in a minimal medium in the presence of *myo*-inositol as a sole carbon source. In 1989, Burtle and Lovell [67] suggested that the liver and intestine of catfish (*Ictalurus punctutus*) synthesize *myo*-inositol *de novo* and hence dietary supplementation is unnecessary. Inositol acquisition by *Cryptococcus neoformans* is perquisite for the successful development of brain infection in animal model [68,69]. The inositol utilization by *Cryptococcus* is considered as most likely factor for the emergence of this pathogen from an environment reservoir [70]. These *myo*-inositol pathways, like L-fucose utilization pathways in *Campylobacter jejuni* [71], could provide a competitive advantage for *A. hydrophila* strains expressing an *iol* cluster to grow in liver, intestine or other fish tissue and could be responsible for enhanced virulence in catfish. Alternatively, the abundance of *myo*-inositol in soil and pond sediments could facilitate growth and survival of *A. hydrophila* isolates, providing a competitive advantage over other bacterial taxa that lack the capacity to utilize *myo*-inositol. Alternatively, the *iolR* transcriptional regulator present within the *iol* cluster may regulate the expression of known *A. hydrophila* virulence factors such as aerolysin [72,73], potentially resulting in *myo*-inositol-dependent expression of multiple VAh genetic loci. 

This study, by sequencing a large number of epidemic and reference *A. hydrophila* isolates followed by the identification of epidemic-associated genomic regions, has provided valuable tools for studying the molecular epidemiology of the ongoing MAS epidemic in the southeastern United States. In this study we found a 100% correlation between presumptive VAh isolates with the presence of specific genomic regions and the ability to use *myo*-inositol as their sole carbon source. In the future, epidemic outbreaks of MAS may be investigated using a genotypic and/or phenotypic assay based on the presence of epidemic-associated genetic loci in epidemic isolates and/or the ability to use *myo*-inositol as a sole carbon source. In addition to routine diagnostics, these assays will also help to track the geographic distribution of the current epidemic *A. hydrophila* strains affecting catfish farming. 

In this study another objective was to attempt to ascertain the origin of this highly virulent genotype. The comparative genomic analysis of VAh and RAh strains identified, in addition to epidemic-associated genomic regions, several other genomic regions that could help trace the emergence of this virulent *A. hydrophila* strain. The lateral transfer of an O-antigen biosynthesis gene cluster is widely considered as a prominent way to generate novel serotypes with highly virulent attributes [74]. In this comparative genomics study we have identified four different novel O-antigen biosynthesis gene clusters among 11 different isolates. These novel O-antigen clusters varied substantially based on their nucleotide sequences, gene content and their relative genetic organization within the clusters. The VAh-type O-antigen cluster, which is present in all sequenced epidemic isolates and one reference isolate TN97-08, is predicted to include the five sugars D-rhamnose, D-mannose, D-Fucose, and 3-acetamido-3, 6-dideoxy-d-galactose (D-Fuc*p*3NAc). Although the genome-wide pairwise comparison between the conserved gene families of ML09-119 and TN97-08 demonstrated that they are less than 75% homologous, it is intriguing to discover that the 26.5 kb O-antigen gene cluster of strain TN97-08, obtained in 1998 from a diseased fish in Tennessee, was 100% identical to that of the recent epidemic *A. hydrophila* isolates. Future experiments will be required to determine whether the novel O-antigen cluster present in epidemic isolates provides any direct role in the virulence of VAh strains in catfish. 

Within the VAh genomes we identified five prophages, four of which were unique to the epidemic strains. Prophage induction using mitomycin C resulted in the maturation of a phage with an icosahedral head and a contractile tail. We isolated DNA from the induced phage and generated phage genome sequences via pyrosequencing. Mapping of the phage genome to the VAh strain ML09-119 genome clearly indicated that prophage AH4 was induced by mitomycin C treatment. Interestingly, we found three putative genes located adjacent to the AH4 prophage that were VAh-associated but not mitomycin C-induced. Their potential role in mediating lysogenic conversion in VAh strains is unknown, and warrants further investigation. 

The expansion of MAS in catfish aquaculture caused by highly pathogenic *A. hydrophila* is threatening the catfish farming industry in the southeastern United States. Currently, there is no effective vaccine or therapeutic agent demonstrated to be effective for the prevention and/or control of MAS in catfish aquaculture ponds. It is our goal to use the molecular insights gained from this comparative genomic analysis to design improved diagnostic and therapeutic approaches for control of epidemic MAS caused by *A. hydrophila*.

## Supporting Information

Dataset S1 **Nucleotide sequences for all contigs greater than 200 bp from the genome of *A. hydrophila* ML09-119.**
(XLSX)

Dataset S2 **Protein sequences of the predicted open reading frames (ORFs) of the *A. hydrophila* genomes sequenced in this study.**
(XLSX)

Dataset S3 **Nucleotide sequences of 55 epidemic-associated unique regions within the genome of VAh strain ML09-119.**
(XLSX)

Dataset S4 **Nucleotide sequences of epidemic-associated unique ORFs predicted within the genome of VAh strain ML09-119.**
(XLSX)

Dataset S5 **Protein sequences of epidemic-associated unique ORFs predicted within the genome of VAh strain ML09-119.**
(XLSX)

Dataset S6 **The BLASTx output of the epidemic-associated ORFs found within the genome of VAh *A. hydrophila* strains.**
(XLSX)

Dataset S7 **Nucleotide sequences of the 16 genomic islands (GIs) found within the genome of VAh strains.**
(XLSX)

Dataset S8 **The BLASTx output of the 16 genomic islands (GIs) found within the genome of VAh strains.**
(XLSX)
